# Predictive Modeling of Mining Laboratory Effluent Contamination Using LSTM-Attention Networks: A Case Study from the Haut-Katanga Copperbelt

**DOI:** 10.1007/s10653-026-03262-0

**Published:** 2026-06-03

**Authors:** Mathieu Kayembe Musala, Matthieu Tshanga Matthieu, Meschac Amani Mugaruka, Fabrice Kalombo Mwambila, Hervé Ngoie Ilunga, Jerry Mbayo Kyongo, Gbenga Olamide Adesola, Arthur Kaniki Tshamala

**Affiliations:** 1 Department of Process Engineering, School of Industrial Engineering, University of Lubumbashi, Lubumbashi, Democratic Republic of Congo; 2 Department of Environmental Sciences, College of Agriculture and Environmental Sciences, University of South Africa, Pretoria, South Africa; 3 School of Mines, Official University of Bukavu, Bukavu, Democratic Republic of Congo; 4 Environmental Analysis Laboratory, Polytechnic Faculty, University of Lubumbashi, Lubumbashi, Democratic Republic of Congo; 5 Department of Industrial Chemistry, Polytechnic Faculty, University of Lubumbashi, Lubumbashi, Democratic Republic of Congo; 6 Department of GIS and Remote Sensing, University of Fort Hare, King Williams, South Africa

**Keywords:** Liquid effluent, Mineral analysis laboratory, Environmental pollution, Time series forecasting, Heavy metals contamination, DRC Mining Code

## Abstract

Mining laboratory effluents in the Democratic Republic of Congo (DRC) represent an undercharacterized source of heavy metal contamination, with direct implications for receiving watershed quality and downstream community health. A comparative time-series modeling framework is applied, incorporating ARIMA as a statistical baseline, alongside Vanilla LSTM and a dual-layer LSTM-Attention architecture, to predict effluent concentrations of pH, suspended solids (SS), and six heavy metals (As, Cu, Fe, Zn, Pb, Ni) from a mineral analysis laboratory in Haut-Katanga. The dataset comprises 43 weekly observations (January to October 2023), with 80% used for training (n = 34) and 20% for testing (n = 9). The LSTM-Attention model demonstrates the highest predictive performance (R^2^ ≈ 0.99 for most parameters; MSE as low as 5.6 × 10⁻^1^⁰ for arsenic), substantially outperforming both ARIMA and Vanilla LSTM, particularly for episodically variable parameters such as copper and iron. Probabilistic forecasts extending to December 2023, incorporating 95% confidence intervals under a Gaussian residual approximation, indicate an elevated risk of regulatory threshold exceedance for SS, Cu, Zn, and Fe, whereas As and Ni are projected to remain within compliant limits. These findings are site-specific and exploratory, given the limited temporal coverage and single-site design. The results highlight the need for targeted pre-treatment interventions, including alkaline neutralization and selective chemical precipitation, and support a transition from reactive compliance monitoring to proactive environmental management in alignment with SDG 6.3 (water quality) and SDG 3.9 (health impacts of pollution).

**Supplementary Information:**

The online version contains supplementary material available at 10.1007/s10653-026-03262-0.

## Introduction

Industrial effluent pollution is widely recognized as a major environmental challenge at the global scale, with mining activities contributing significantly to the degradation of aquatic ecosystems and posing risks to public health. It has been estimated that mining operations worldwide discharge approximately 1.5 billion cubic meters of contaminated water each year, which often contains high concentrations of heavy metals, acidic compounds, and suspended solids (Zhang et al., 2021). While a considerable number of studies have investigated large-scale mining discharges, particularly acid mine drainage and tailings-related pollution in industrialized regions such as North America, Australia, and Europe, there remains a notable lack of research focusing on laboratory-scale effluents in developing countries (DONGO et al., 2013; Omisore, 2018).

Recent investigations have shown that mineral analysis laboratories, although operating at much smaller volumes compared to large processing plants, can generate highly concentrated waste streams containing complex mixtures of heavy metals, including Cu, Fe, Zn, Pb, As, and Ni, as a result of intensive acid digestion procedures and the use of analytical reagents (Nwobi et al., 2025). In Sub-Saharan Africa, where mining activities contribute between 15 and 30% of national GDP in several countries, such laboratory effluents are often discharged with limited or no treatment, leading to direct impacts on surface water quality and downstream communities (Kalala et al., 2016). The Haut-Katanga Province in the Democratic Republic of Congo, which hosts one of the world’s most significant copper-cobalt mining districts, represents a clear example of this situation, where the rapid expansion of mineral analysis laboratories has not been matched by systematic environmental monitoring or predictive assessment of their impacts (Cailteux et al., 2005).

Conventional monitoring of effluents in mining environments is commonly based on descriptive statistics, compliance checks against regulatory thresholds, or the application of classical regression models (Su et al., 2025). Although these approaches can provide useful information on water quality conditions at specific time points, they present several important limitations that reduce their effectiveness for environmental protection purposes. Firstly, conventional methods are generally unable to capture the non-linear and time-dependent nature of pollutant dynamics. Parameters such as heavy metal concentrations, pH, and suspended solids often exhibit complex temporal behavior influenced by laboratory working schedules, variations in analytical workload, and reagent consumption, which cannot be adequately represented using linear models (Al-Bahadili & Drewil, 2022). Secondly, threshold-based compliance monitoring is essentially reactive, as exceedances are only identified after they have already occurred, meaning that environmental impacts may take place before corrective actions are implemented (Malashin et al., 2024). Third, classical statistical approaches have limited capacity to account for delayed responses and memory effects in effluent quality, where current discharge conditions depend on historical operational activities over extended periods (Korunoski, 2019; Liu et al., 2025). These limitations indicate the need for more advanced modelling approaches that are capable of learning complex temporal patterns directly from historical data and that can support proactive environmental management strategies.

Time series modelling has become an increasingly important tool for analyzing pollution dynamics in liquid effluents, as many water quality parameters exhibit strong temporal variability (Al-Janabi et al., 2020; Runge & Zmeureanu, 2021). The Autoregressive Integrated Moving Average (ARIMA) model is one of the most commonly applied traditional approaches in environmental time series analysis and is effective in representing linear temporal trends (Yetilmezsoy & Saral, 2007). However, ARIMA models rely on assumptions of stationarity and linearity, which limit their applicability in situations where pollution dynamics are influenced by non-linear interactions and long-term dependencies (Da Silva et al., 2025).

In recent years, deep learning techniques, particularly Long Short-Term Memory (LSTM) neural networks, have been increasingly applied in environmental prediction studies. LSTM models are designed to capture long-term dependencies in sequential data through memory cells and gating mechanisms, which makes them suitable for modelling the complex temporal behavior observed in pollution datasets (Chen et al., 2018). Leščešen et al. (2025) and Gandhi et al. (2024) found that LSTM-based models beat ARIMA in river flow and water quality prediction. Attention mechanisms in LSTM designs allow the network to prioritize past data, improving model performance. Environmental applications where operational periods or episodic pollution occurrences significantly affect effluent quality benefit from this characteristic (Wen & Liu, 2023).

Compared to existing studies on effluent quality prediction, the present work differs in three important respects. Previous applications of ARIMA to water quality time series (Yetilmezsoy & Saral, 2007) demonstrated its value for smooth, stationary trends but did not address laboratory-scale batch discharges or integrate regulatory threshold analysis into the forecast output. Applications of pure LSTM models to environmental series (Chen et al., 2018; Gandhi et al., 2024) improved predictive accuracy but rarely embedded confidence-interval-based compliance risk. The LSTM-Attention architecture applied here combines the temporal selectivity of the attention mechanism with dual-layer depth to handle the episodic, operationally-driven contamination patterns specific to mineral analysis laboratories. Furthermore, integration of the DRC Mining Regulations directly into the forecast hazard classification transforms statistical predictions into compliance-relevant outputs, which represents a methodological advance beyond prior environmental forecasting studies in the region.

The present study addresses the identified research gaps by developing and validating a predictive modelling framework for analyzing temporal contamination dynamics in liquid effluents generated by a mineral analysis laboratory in the Haut-Katanga Province. The specific objectives of the study are to (i) characterize the physicochemical composition of laboratory effluents and assess historical compliance with DRC Mining Regulations for pH, suspended solids, and selected heavy metals; (ii) implement and compare the performance of ARIMA, Vanilla LSTM, and dual-layer LSTM-Attention models in predicting effluent quality; (iii) generate short-term forecasts incorporating 95% confidence intervals in order to evaluate the probability of regulatory exceedances; and (iv) propose practical recommendations for pre-treatment and operational improvements to reduce environmental risks.

The originality of this work rests on three specific methodological contributions that advance the state of the art beyond simply applying an existing architecture to a new dataset. First, whereas prior studies in Sub-Saharan African mining contexts have reported static compliance checks or basic regression models (Dongo et al., 2013; Kalala et al., 2016), this study presents an end-to-end pipeline that integrates multi-parameter effluent preprocessing, sequence modelling, attention-weight visualisation, and probabilistic non-compliance analysis within a single reproducible framework. Second, this is the first application of a dual-layer LSTM-Attention architecture to mining laboratory-scale effluents in the DRC Copperbelt, a context distinct from both industrial-scale wastewater treatment plants and large open-pit tailings impoundments studied elsewhere. Third, the integration of 95% confidence interval forecasts with DRC regulatory thresholds, combined with the development of a parameter-specific risk prioritization matrix, enables the translation of model outputs into actionable decision-support information for laboratory managers and regulators. This approach extends beyond conventional accuracy-focused benchmarking studies. Collectively, these contributions support a shift from reactive threshold monitoring toward proactive, data-driven environmental management.

## Materials and methods

### Study area

**Figure. 1:**
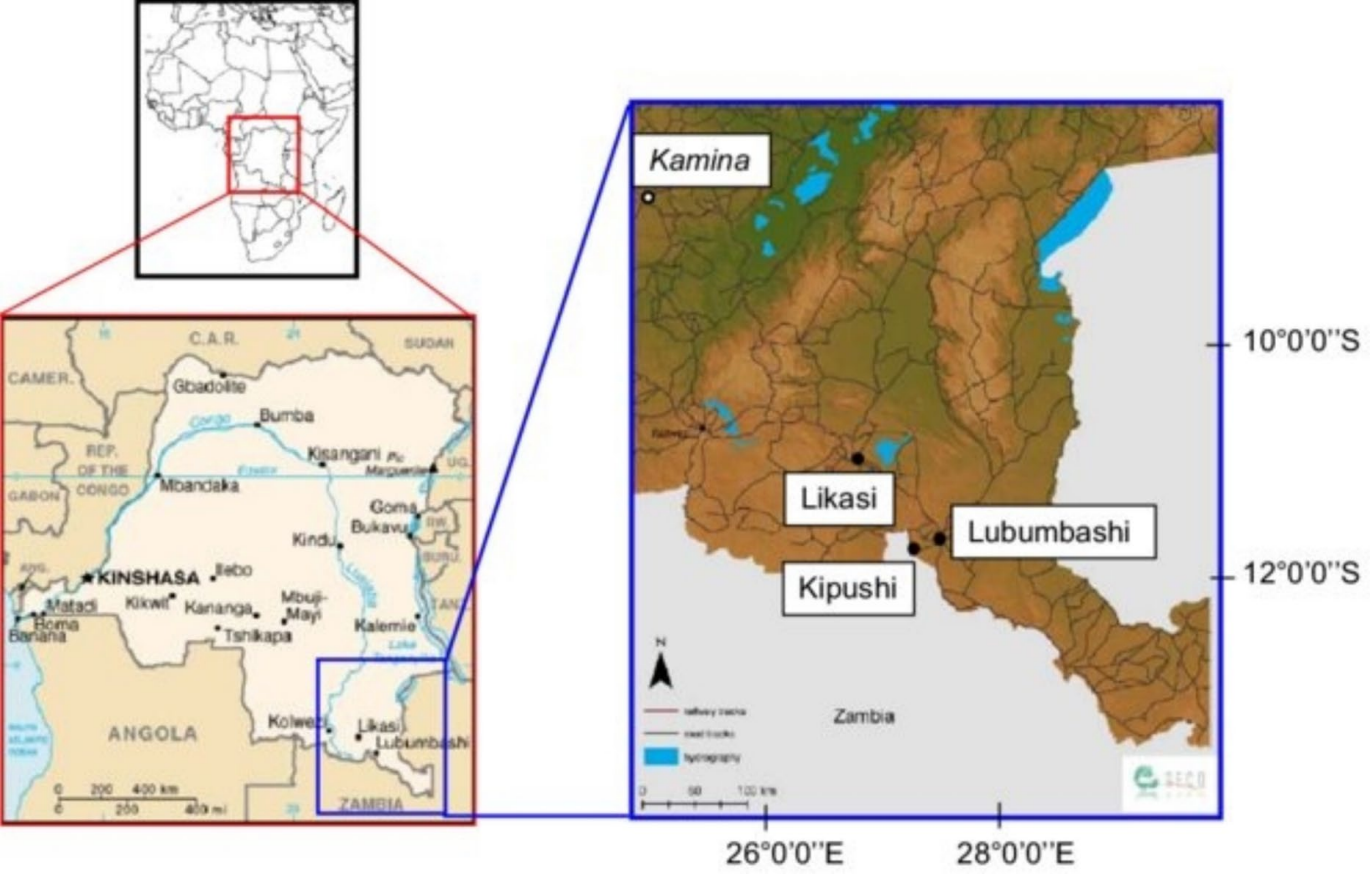
Locality map of the study area with an insert map of the Democratic Republic of Congo (DRC)

The study area is located in the Upper Katanga Province, in the eastern part of the Democratic Republic of the Congo (Fig. 1). The area hosts a high concentration of mineral processing and analysis facilities, reflecting its strategic importance within the national mining sector. The upper Katanga lies between latitudes 08° 55′ 0″ S and 12° 48′ 0″ S and longitudes 25° 43′ 0″ E and 29° 48′ 0″ E, respectively. The study area experiences tropical savanna climate, with the rainy season starting in October and ending in March the next year and accounting for 92% of rainfall throughout the year, while the dry period starts from April to September. The average annual precipitation is roughly 1,860 mm, and the mean temperatures range from 21 °C to 35 °C. At an average speed of 5.8 m/s and a maximum speed of 15 m/s, the northeast and southeast wind directions are the most common (Basson et al., 2022; Van Langendonck et al. 2013).

### Geological context

The study region is located in the Neoproterozoic Katangan Supergroup (Lufilian Arc) in the southeastern DRC (Ali & Alshammari, 2021; Delpomdor et al., 2018). This supergroup in central Africa reaches from northern Zambia to the southeastern Democratic Republic of the Congo. It is approximately 10 km thick and is split into three sections based on the existence of two regional diamictites: Roan (R), Nguba (Ng), and Kundelungu (Ku) from bottom to top. The Lufilian Arc and related fold-thrust belt formed during the Lufilian Orogeny, caused by the convergence and collision of the Congo and Kalahari cratons between 750 and 550 Ma. This orogenic band has world-class stratiform copper-cobalt deposits that have sparked decades of geological research and exploration since the early twentieth century (Kipata et al. 2013). The exposed segment of the Lufilian Arc is bordered to the southeast by the Archean-Paleoproterozoic Bangweulu block, to the east by the Paleoproterozoic Ubende belt, and to the west by the Mesoproterozoic Kibaran belt. To the north and northwest, the Katangan Supergroup features a basal unconformity that covers the Kibaran basement. The inner and middle Luflian also have a basement (Paleoproterozoic or Mesoproterozoic).

### Sampling and data collection

#### Sampling protocol

Final liquid effluent samples were systematically collected from the discharge point of the mineral analysis laboratory treatment system. Samples were collected using pre-cleaned high-density polyethylene (HDPE) bottles with a capacity of 1 L, which were previously acid-washed with 10% HNO_3_ and rinsed three times with deionized water in order to avoid contamination. Two different sampling approaches were applied depending on the accessibility of the discharge infrastructure. When a sampling port was available, effluent was collected directly from the discharge valve after allowing the system to flush for approximately 30 s to ensure that representative samples were obtained. In cases where sampling was carried out from the neutralization tank, where effluent is treated with caustic soda (NaOH), a peristaltic pump was used to collect samples from mid-depth in order to avoid surface films and settled solids (Muniz et al., 2025; Shakhsher et al., 2014).

All collected samples were labelled with unique identifiers indicating the sampling date, time, and location and were transported to the analytical laboratory in coolers maintained at approximately 4 °C. Analyses for pH and suspended solids were initiated within 24 h, while heavy metal analyses were carried out within 48 h, in accordance with standard preservation procedures.

#### Sampling frequency

The sampling frequency was defined in accordance with Article 73 of Annex VIII of the DRC Mining Regulations (Decree n° 038/2003, as amended by Decree n° 18/024 of June 8, 2018). The adopted sampling schedule included pH and suspended solids measurements three times per week, specifically on Mondays, Wednesdays, and Fridays, with samples collected between 10:00 and 11:00 AM to reduce temporal variability related to daily operational changes. Heavy metals were analyzed once per week using a composite sample prepared by combining equal volumes from the three weekly samples.

This sampling strategy generated a dataset covering the period from January to October 2023, which provided sufficient temporal resolution to capture both short-term variations and longer-term trends in effluent quality.

This sampling strategy generated a dataset of n = 43 weekly observations (January–October 2023). pH and suspended solids, measured three times per week (Mondays, Wednesdays, and Fridays), were averaged to a single weekly value before modelling. Composite metal samples (prepared from equal volumes of the three weekly aliquots) were used directly as one observation per week. Table S2 (Supplementary Material) summarises for each parameter: raw measurement frequency, total raw observations, percentage of missing values (range: 0–4.7%), and number of outliers removed by interquartile range (IQR) filtering.

### Physicochemical analysis

All physicochemical analyses were conducted using standard procedures to ensure data quality and consistency (Aniyikaiye et al., 2019).

#### Suspended solids

Suspended solids concentrations were measured using a HACH DR/890 portable colorimeter for photometric analysis. This method relies on how light diminishes as it passes through the sample, which correlates with the concentration of suspended particles. The instrument was daily calibrated with certified formazin turbidity standards that matched the expected concentration range. Samples that went beyond the calibration range were diluted with deionized water, and the results were adjusted for the dilution factor. The method’s detection limit was 5 mg/L, with a relative standard deviation of about ± 3% (Carvalho & Tanner, 2007; Rouessac et al., 2004).

#### pH and other parameters

pH and other physicochemical parameters were measured with a HANNA HI 9829 portable meter. The instrument measured pH, electrical conductivity, dissolved oxygen, oxidation–reduction potential, and temperature all at once. The pH electrode was calibrated with certified buffer solutions (pH 4.01, 7.00, and 10.01) using a three-point procedure before each sampling session. Measurements were taken at room temperature with automatic temperature compensation on, and pH values were recorded after the electrode stabilized (Friedler et al., 2021; Salari et al., 2025).

#### Heavy metal analysis

Heavy metal concentrations (As, Cu, Fe, Zn, Pb, and Ni) were measured using a PG Instruments AA500 atomic absorption spectrometer in both flame and graphite furnace modes. Before analysis, samples were acidified to a pH of less than 2 with concentrated HNO_3_. For samples with visible particles, we performed acid digestion and then filtered through 0.45 μm membranes. Calibration applied certified multi-element standards, and quality control involved analyzing blanks, duplicates, and spiked samples. Acceptable analytical performance was defined as spike recoveries between 90 and 110%, with relative percent differences under 10% (Yin et al., 2025).

### Determination of flow rates (quantities)

The effluent discharge flow rate (*d*, in m^3^/h) was calculated by measuring storage tank water level fluctuations over time. The volumetric flow rate was the volume difference between two measurements divided by time. Ultrasonic level sensor readings and tank dimensions were utilized to determine tank volume, assuming cylindrical shape (Moayedi et al., 2024; Zhang, 2022):

1 $$ d = \left( {V\_2 - V\_1} \right)/\left( {t\_2 - t\_1 } \right) $$

$V\_2 - V\_1$: The difference in volume represents the quantity of final liquid effluent produced.

$t\_2 - t\_1$: Time interval: generally, 48 h between the first and second measurement.

Volumes were calculated based on the tank’s cylindrical geometry (*V* = *πr*^*2*^* h*).

This approach provides sufficient accuracy for operational monitoring and for use in time-series modelling, although it does not capture short-term flow fluctuations.

### Data preprocessing

To prepare raw analytical data for modelling, they were cleaned, normalized, temporally aggregated, and stationarized (Patthi et al., 2024). To differentiate between real occurrences and measurement errors, outliers were found using the interquartile range approach and compared to laboratory records. Missing values were addressed using interpolation or forward-filling, depending on gap length. Min–Max normalization scaled the variables to the [0, 1] range via parameters only from the training dataset to prevent data leakage. Data were combined into weekly intervals for consistency across parameters (Wang et al., 1993; Yin et al., 2025).

Normalization followed the Min–Max transformation

2 $$ x\_norm = \left( {x - x\_\min } \right)/\left( {x\_\max - x\_\min } \right) $$

### Modeling framework

A comparative modelling framework was implemented, consisting of one classical statistical model and two deep learning models (Aniyikaiye et al., 2019; Carvalho & Tanner, 2007).

#### ARIMA model

An ARIMA model was selected as a classical statistical baseline to provide a reference point for quantifying the added value of deep learning architectures. It is important to note that ARIMA and the LSTM-based models are not methodologically equivalent competitors: ARIMA requires differenced, stationary input series and assumes linearity and Gaussian residuals, while LSTM-Attention operates directly on raw sequences and can model non-linear temporal dependencies. The comparison therefore aims to illustrate the incremental gain from deep learning, not to establish a fair head-to-head contest between structurally different model families. For each monitored parameter, the optimal ARIMA order (p, d, q) was identified independently by searching p, q ∈ {0, 1, 2, 3} and d ∈ {0, 1, 2} and selecting the specification that minimised the Akaike Information Criterion (AIC). Although ARIMA (1,1,1) emerged as optimal for most parameters, parameter-specific orders are reported in Table S1 (Supplementary Material). Model implementation used the statsmodels library in Python.

The ARIMA model is expressed as
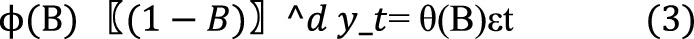


[Display Image Removed]where p: Order of the autoregressive component (number of lag observations); d: Degree of differencing required to achieve stationarity and q: Order of the moving average component,

#### Vanilla LSTM model

A standard Long Short-Term Memory network was implemented with a single LSTM layer followed by a dense output layer. The model used a lookback window of 12 weeks and was designed to capture temporal dependencies in the effluent quality data (Kumar, 2023; Vatsa, 2024).

4 $$ f\_\left( {t } \right) = \sigma \left( {W\_\left( {f } \right).\left[ {h_{t - 1 } ,x_{t } } \right] + b_{f} } \right) $$

5 $$ i_{t } = \sigma \left( {W_{i } .\left[ {h_{t - 1 } ,x_{t } } \right] + b_{i} } \right) $$

6 $$ C\sim_{t } = \tanh \left( {W_{c } .\left[ {h_{t - 1 } ,x_{t } } \right] + b_{c} } \right) $$

7 $$ C_{t } = f_{t } *C_{t - 1 } + i_{t } *C\sim_{t } $$

8 $$ o_{t } = \sigma \left( {W_{0 } .\left[ {h_{t - 1 } ,x_{t } } \right] + b_{0} } \right) $$

9 $$ h_{t } = o_{t } * tanh\left( {C_{t } } \right) $$

where σ denotes the sigmoid activation function, tanh is the hyperbolic tangent function, ∗ represents element wise multiplication, $W_{f } ,W_{i} ,W_{c} ,W_{o}$, ​ are the weight matrices, $b_{f} ,b_{i} ,b_{c} ,b_{o}$ are the bias vectors, $x_{t }$ is the input vector, $h_{t }$ is the hidden state, and $C_{t }$ is the cell state at time stept.

#### LSTM-Attention Model

The LSTM-Attention architecture consisted of two stacked LSTM layers followed by an attention mechanism, which enabled the model to assign different weights to historical time steps when generating predictions (Zhang et al., 2021).

10 $$ e_{t } = v^{T} tanh\left( {W_{h } ,h_{t } + b_{h} } \right) $$

11 $$ \alpha_{t } = \frac{{\exp \left( {e_{t } } \right)}}{{\mathop \sum \nolimits_{k = 1}^{T} \exp \left( {e_{k } } \right)}} $$

12 $$ C = \mathop \sum \limits_{k = 1}^{T} \alpha_{t } h_{t } $$

where $h_{t }$ represents the hidden states produced by the LSTM layers, $W_{h }$​, v, and $b_{h}$​ denote learnable parameters of the attention mechanism, and $C$ is the context vector computed as a weighted sum of the hidden states. This context vector summarizes the most relevant temporal information and is subsequently used for prediction.

#### Training parameters

All neural networks were implemented in PyTorch and optimised using hyperparameters selected through a structured grid search over learning rate α ∈ {0.001, 0.005, 0.01}, epochs ∈ {50, 100, 150, 200}, and batch size ∈ {8, 16, 32}. The configuration minimising validation loss was retained. The lookback window of 12 weeks was chosen to capture approximately three months of temporal patterns, meaningful for mining laboratory quarterly production cycles. Final parameters (Koblan et al., 2023; Moayedi et al., 2024):

Learning Rate (α): 0.00854 (Adam Optimizer).

Learning Rate (α): 0.00854 (Adam Optimizer).

Weight Update

13 $$ w_{t + 1} = w_{t} - \alpha \frac{\partial L}{{\partial w_{i} }} $$

Epochs / Batch Size: 100 / 16.

Sequence Length (Look-back): 12 observation points.

Loss Function ($L$): Mean Squared Error (MSE).

## Forecasting and visualisation

The models employed a recursive multi-step forecasting strategy (Sun, 2022) to project concentrations for a horizon of **20 steps** (*h* = 20):

14 $$ \hat{y}_{t + h} = f\left( {y_{t + h - 1} , \ldots ,y_{t + h - 12} } \right) $$

Results were visualized using consolidated plots (Korunoski, 2019) showing historical data, hindcasting fit, and future projections. 95% Confidence Intervals were calculated as 1.96σ of the residuals. This represents a first-order probabilistic approximation under a Gaussian residual assumption. Shapiro–Wilk tests applied to LSTM-Attention residuals showed no statistically significant departure from normality for most parameters (p > 0.05), providing partial empirical support for this assumption. Alternative distribution-free approaches such as bootstrap resampling or quantile regression are recommended for future work. Final dangerousness was assessed by superimposing DRC Regulatory Limits (Lreg) onto the forecast (Zheng et al., 2025):

15 $$ Hazard Status = \left\{ {\begin{array}{*{20}c} {Critical if \hat{y}_{t + h} > L_{reg} } \\ {Compliant otherwise} \\ \end{array} } \right. $$

## Model Evaluation Metrics

Training and Validation Loss: Loss allows us to detect overfitting or underfitting (Moayedi et al., 2023; Nwobi et al., 2025):

$$ L_{train } et L_{val } $$

Mean Squared Error (MSE) (Chicco & Jurman, 2021).

16 $$ MSE = \frac{1}{N}\mathop \sum \limits_{i = 1}^{N} \left( {y_{i} - \hat{y}_{i} } \right)^{2} $$

Mean Absolute Error (MAE): is another measure of absolute error on predictions:

17 $$ MSE = \frac{1}{N}\mathop \sum \limits_{i = 1}^{N} \left( {y_{i} - \hat{y}_{i} } \right)^{2}$$

Coefficient of Determination (R^2^): Measures the proportion of variance explained by the model (Al-Bahadili & Drewil, 2022):

18 $$ R^{2} = 1 - \frac{{\mathop \sum \nolimits_{i = 1}^{N} \left( {y_{i} - \hat{y}_{i} } \right)^{2} }}{{\mathop \sum \nolimits_{i = 1}^{N} \left( {y_{i} - \hat{y}_{i} } \right)^{2} }} $$

where $\hat{y}$ is the mean of the actual values.

Residual Analysis: Residuals $r_{i}$ are (Patthi et al., 2024).

19 $$ r_{i} = y_{i} - \hat{y}_{i} $$

After calculating residuals, they are analyzed to detect any bias in the predictions (Yin et al., 2025)

20 $$ {\text{Variance of Residuals}} = \frac{1}{N}\mathop \sum \limits_{i = 1}^{N} r_{i}^{2} $$

## Results

### Baseline physicochemical characterization

#### Suspended solids and pH

**Figure. 2:**
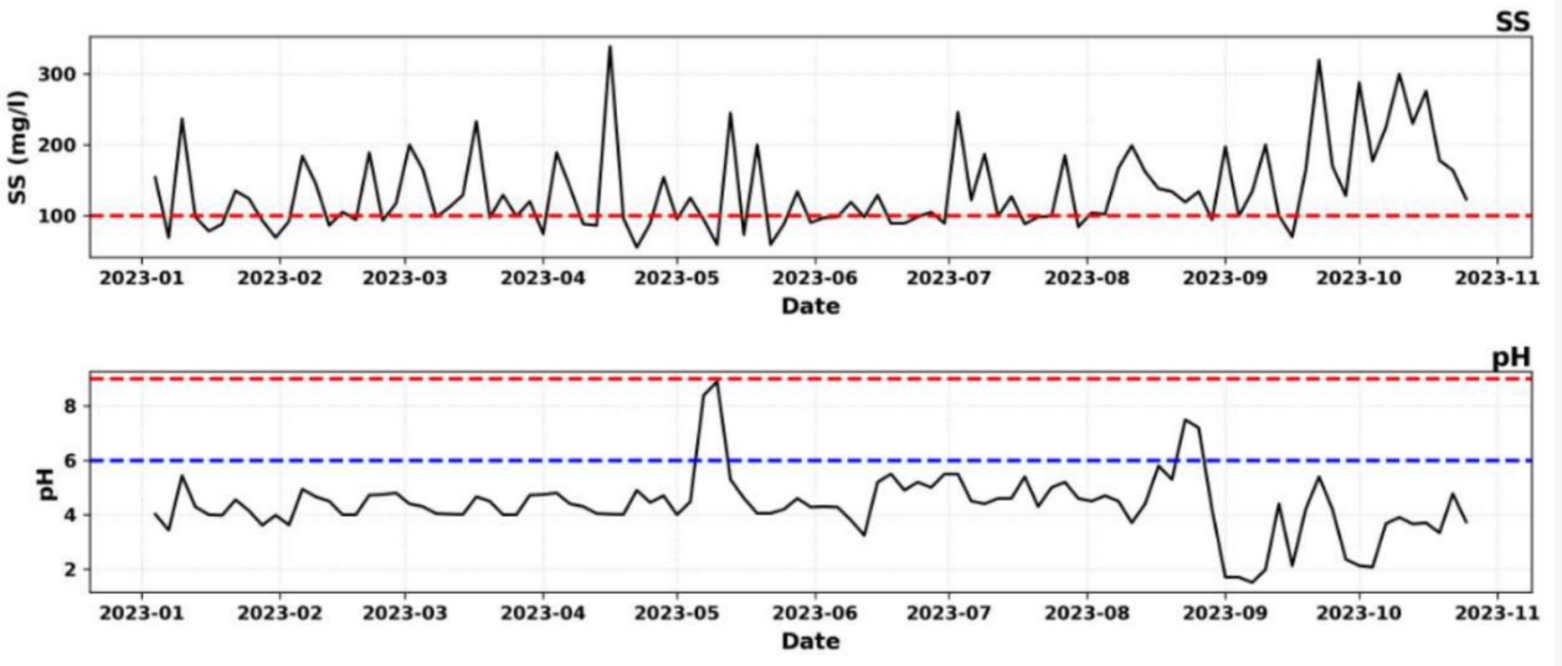
Evolution of effluent physiochemical composition from January to November 2023, showing historical series against min (blue line)/max (red line) thresholds

The physicochemical characterization of the laboratory liquid effluents indicates notable deviations from the regulatory standards established under the DRC Mining Regulations (Decree n° 038/2003, as amended by Decree n° 18/024). Monthly average concentrations of suspended solids (SS) and pH values recorded over the 10-month monitoring period (January–October 2023) are presented in Table 1, while Fig. 2 illustrates the temporal evolution of these parameters in relation to the applicable regulatory limits (Da Silva et al., 2025; Malashin et al. 2024).

**Table 1:** Annual physicochemical (pH, SS) values

Parameter	January	February	March	April	May	June	July	August	September	October	Average	Ref.MR
SS(mg/l)	115	123	138	131	115	101	134	135	158	218	136.7	100
pH	4.15	4.44	4.265	4.436	5.17	4.692	4.81	5.18	2.96	3.44	4.35	6–9

^*^Ref.: reference MR: Mining Regulation of the DRC

The suspended solids results show that concentrations exceeded the regulatory limit of 100 mg/L in eight out of ten monitored months, with June showing marginal compliance at 101 mg/L, which falls within the range of analytical uncertainty. The annual average concentration of 136.7 mg/L represents an exceedance of approximately 37% above the permissible limit. A clear increasing trend is observed toward the end of the monitoring period, during September (158 mg/L) and October (218 mg/L), suggesting a progressive decline in treatment efficiency or an increase in laboratory operational activity during these months (Carvalho & Tanner, 2007; Muniz et al., 2025).

Suspended solids present in laboratory effluents originate from several sources, including precipitated metal hydroxides formed during neutralization processes, undissolved mineral residues from sample digestion, and colloidal silica resulting from silicate dissolution. Elevated SS concentrations are of environmental concern, as particulate matter can act as a carrier for adsorbed heavy metals, organic compounds, and other pollutants, thereby increasing contaminant transport and toxicity in receiving water bodies (Friedler et al., 2021).

The pH results reveal a more severe compliance issue. All monthly average pH values are substantially below the minimum regulatory threshold of 6.0, with values ranging from 2.96 to 5.18. The annual mean pH of 4.35 indicates persistent acidic conditions throughout the year. The lowest pH values were recorded in September (2.96) and October (3.44), coinciding with the highest suspended solids concentrations, which suggests a possible relationship between increased laboratory activity and deteriorating effluent quality.

This persistent acidity is attributed to several contributing factors, including residual mineral acids (HNO_3_, HCl, and H_2_SO_4_) used in aqua regia digestion of geological samples, the addition of acidic preservatives to maintain metal solubility during storage, and insufficient neutralization capacity within the treatment system (Friedler et al., 2021; Koblan et al., 2023). Acidic effluent discharge poses serious environmental risks, including mobilization of heavy metals from sediments, disruption of aquatic pH buffering systems, and direct physiological stress to aquatic organisms, even under diluted conditions (Muniz et al., 2025).

Figure 2 demonstrates the temporal variation of SS and pH relative to their regulatory thresholds, clearly showing systematic pH violations and an intensifying trend of suspended solids exceedances over time. These findings highlight the need for immediate corrective measures and support the application of predictive modeling approaches adopted in this study.

#### Chemical composition

**Figure. 3:**
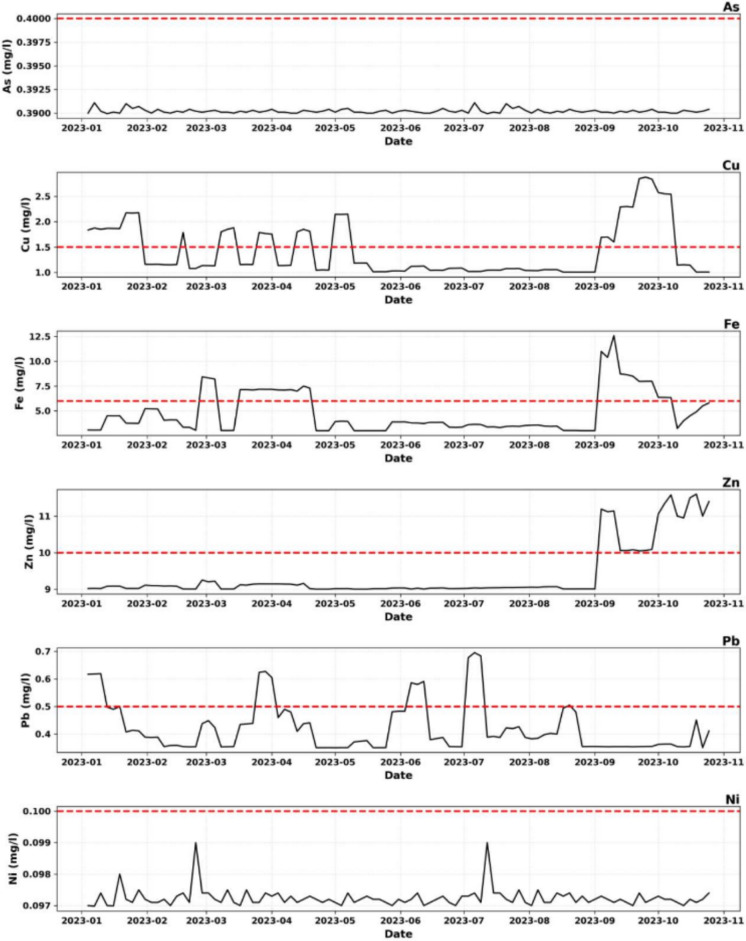
Evolution of the effluent chemical composition from January to November 2023, showing Historical Series against the max threshold

The chemical composition (As, Cu, Fe, Zn, Pb, Ni) of the effluents is presented synthetically in the following lines: Values obtained from January to October are presented in Table 2, and the evolution is illustrated in Fig. 3 (Yao et al., 2023; Yetilmezsoy & Saral, 2007).

**Table 2:** Annual chemical composition (As, Cu, Fe, Zn, Pb,Ni) values

Parameter	January	February	March	April	May	June	July	August	September	October	Average	Ref.MR
As (mg/l)	0.390	0.39	0.39	0.39	0.39	0.39	0.39	0.39	0.39	0.39	0.39	0.4
Cu (mg/l)	1.89	1.21	1.48	1.38	1.37	1.08	1.05	1.03	2.15	1.57	1.42	1.5
Fe (mg/l)	3.91	4.53	6.14	5.93	3.42	3.67	3.47	3.26	8.69	5.22	4.82	6
Zn (mg/l)	9.38	9.38	9.38	9.09	9.01	9.02	9.04	9.03	10.28	11.27	9.49	6
Pb (mg/l)	0.49	0.37	0.45	0.44	0.38	0.45	0.49	0.42	0.36	0.37	0.42	0.5
Ni (mg/l)	0.09	0.10	0.09	0.09	0.09	0.09	0.09	0.09	0.09	0.09	0.10	1

^*^Ref.: reference MR: Mining Regulation of the DRC

##### Arsenic (As)

Arsenic concentrations remained constant at 0.390 mg/L throughout the monitoring period, approaching but not exceeding the regulatory limit of 0.4 mg/L. This stability, corresponding to 97.5% of the permissible limit, suggests either a consistent low arsenic content in the analyzed mineral samples or effective retention and precipitation during treatment. The absence of temporal variability is unusual for laboratory effluents and may indicate that arsenic is present predominantly as dissolved arsenate species at relatively stable background concentrations (Yao et al., 2023). Although compliant, the narrow safety margin necessitates continued monitoring.

##### Copper (Cu)

Copper exhibited the greatest variability among the monitored metals, with concentrations ranging from 1.03 mg/L in August to 2.15 mg/L in September. Regulatory exceedances were observed in January, September, and October. Although the annual average concentration (1.42 mg/L) remains below the regulatory limit, this masks frequent short-term violations evident in higher-resolution sampling data.

Copper concentration trends are closely linked to laboratory analytical activity, particularly during intensive digestion of copper-rich samples typical of the Katangan Copperbelt, where copper sulfide and oxide minerals dominate. Periods of elevated copper concentrations coincide with increased analytical demand, especially toward the end of the dry season when mining companies intensify grade control analyses (Friedler et al., 2021; Su et al., 2025). Environmentally, copper is of concern due to its toxicity at relatively low concentrations, where it disrupts photosynthesis and osmoregulation and bioaccumulates in aquatic organisms (Dai et al., 2022).

##### Iron (Fe)

Iron concentrations showed pronounced temporal variability, with values ranging from 3.26 mg/L to 8.69 mg/L. Regulatory exceedances occurred in March and September, with the September peak representing a significant pollution event. Iron inputs originate from acid digestion of iron-bearing minerals, corrosion of metallic laboratory equipment, and potential use of iron-based coagulants. The temporal correspondence between iron and copper peaks suggests common operational drivers linked to sample processing intensity (Malashin et al., 2024).

While iron is less toxic than some heavy metals, elevated concentrations cause aesthetic degradation, promote bacterial iron oxidation, and influence the behavior and bioavailability of other contaminants (Muniz et al., 2025; Olascoaga & Haller, 2012).

##### Zinc (Zn)

Zinc concentrations remained relatively stable for most of the monitoring period before increasing in September and October. Although the annual average remained within regulatory limits, the late-period upward trend indicates declining treatment performance or increased zinc input. Zinc presence reflects the sphalerite-rich nature of regional ore deposits, and elevated concentrations pose ecological risks due to chronic toxicity to aquatic organisms (Chen et al., 2018).

##### Lead (Pb)

Lead concentrations remained below the regulatory limit throughout the monitoring period; however, several values were close to the threshold, indicating fragile compliance. Occasional exceedances observed in higher-resolution sampling data suggest episodic contamination during galena analysis. Given lead’s cumulative toxicity and lack of a safe exposure threshold, even near-limit concentrations are environmentally and public-health relevant (Hu et al., 2025; Moayedi et al., 2024).

##### Nickel (Ni)

Nickel concentrations were consistently low and well below the regulatory limit, reflecting lower nickel content in regional ores and effective removal during neutralization. Current nickel levels pose minimal environmental risk, though routine monitoring remains necessary to detect potential changes in operational conditions (Jadhav & Pawar, 2023).

#### Synthesis of baseline characterization

The baseline assessment identifies a dual-tier contamination profile. Critical parameters (pH, SS, Cu, Fe, Zn) show frequent violations or narrow safety margins, while As, Pb, and Ni remain generally compliant but require continued monitoring. The strong temporal association between contamination peaks and laboratory operational intensity confirms that effluent quality is operationally driven rather than random. This observation supports the application of time-series forecasting to anticipate future contamination trends and inform proactive environmental management (Wen & Liu, 2023).

### Predictive model performance evaluation

#### Model architecture performance

In this study, three different modelling approaches were implemented and comparatively evaluated, including a statistical baseline model (ARIMA), a conventional deep learning model (Vanilla LSTM), and a newly developed hybrid architecture (dual-layer LSTM-Attention). All of the models were trained using the same datasets and were evaluated using identical performance metrics in order to ensure consistency and fairness in the comparison process. Table 3 presents the detailed performance metrics obtained for each model across the eight monitored parameters considered in this study.

**Table 3:** Model Performance

Variable	Model	MSE	MAE	R^2^
As	LSTM-Attention	0.00000000056	0.000018	0.99
	Vanilla-LSTM	0.00000000073	0.000021	0.98
	ARIMA	0.000000051	0.00017	-0.31
Cu	LSTM-Attention	0.0012	0.03	0.99
	Vanilla-LSTM	0.0041	0.049	0.98
	ARIMA	0.098	0.18	0.63
Fe	LSTM-Attention	0.036	0.15	0.99
	Vanilla-LSTM	0.071	0.20	0.99
	ARIMA	2.1	0.66	0.57
SS	LSTM-Attention	54	6.0	0.99
	Vanilla-LSTM	59	5.7	0.99
	ARIMA	3400	43	0.084
Ni	LSTM-Attention	0.0000000018	0.000031	0.99
	Vanilla-LSTM	0.0000000030	0.000042	0.97
	ARIMA	0.00000010	0.00018	-0.14
Pb	LSTM-Attention	0.000058	0.0059	0.99
	Vanilla-LSTM	0.00016	0.0087	0.98
	ARIMA	0.0047	0.032	0.36
Zn	LSTM-Attention	0.0019	0.035	0.99
	Vanilla-LSTM	0.0045	0.05	0.99
	ARIMA	0.093	0.10	0.85
pH	LSTM-Attention	0.024	0.12	0.97
	Vanilla-LSTM	0.038	0.14	0.97
	ARIMA	0.90	0.63	0.37

##### ARIMA model performance analysis

The ARIMA (1,1,1) model, which was used as the baseline statistical approach, shows acceptable performance only for parameters that exhibit relatively smooth and stable temporal trends, such as zinc (R^2^ = 0.85). However, the model performs poorly for parameters characterised by high temporal variability. In particular, ARIMA yields negative R^2^ values for arsenic (− 0.31) and nickel (− 0.14), which indicates that the model performs worse than a simple mean-based prediction, reflecting a fundamental limitation in its predictive capability (Wang et al., 1993, 2018).

For pH, the ARIMA model achieves an R^2^ value of only 0.37 with a mean absolute error of 0.63 pH units, which represents an unacceptable level of error given that regulatory compliance is defined within a relatively narrow range of 6.0 to 9.0. Similarly, the very high MSE value observed for suspended solids (3400mg^2^/L^2^) reflects the inability of the ARIMA model to capture the sharp concentration peaks that occur during September and October.

These limitations can be attributed to the core assumptions underlying the ARIMA framework, including linearity, data stationarity (or difference stationarity), and the independence of residual errors. In the context of laboratory effluent quality, these assumptions are frequently violated, as relationships between variables are often nonlinear, non-stationary behavior persists even after differencing due to operational regime changes, and contamination events tend to exhibit temporal persistence. While ARIMA remains useful for baseline trend identification and interpretability, it is clearly insufficient as a primary forecasting tool for complex wastewater quality parameters (Da Silva et al., 2025; Yao et al., 2023).

##### Vanilla LSTM performance analysis

The Vanilla LSTM model demonstrates a substantial improvement in predictive performance when compared to the ARIMA baseline across all monitored parameters. The model achieves R^2^ values ranging between 0.97 and 0.99, accompanied by significantly lower MSE and MAE values. For arsenic, the LSTM records an MSE of 7.3 × 10^−10^ compared to ARIMA’s 5.1 × 10^−8^, representing an improvement of approximately 70 times. Comparable improvements are observed for copper, iron, and lead.

The improved performance of the LSTM model can be attributed to its architectural characteristics, including memory cells that retain information over extended temporal sequences, nonlinear activation functions that allow complex variable interactions to be modelled, and effective gradient flow mechanisms that reduce vanishing gradient problems during backpropagation (Chen et al., 2018). In this study, the use of a 12-week lookback window enabled the LSTM to capture longer-term dependencies in effluent quality dynamics.

For suspended solids, the Vanilla LSTM reduces the MAE to 5.7 mg/L compared to 43 mg/L for ARIMA, indicating a substantial improvement in practical prediction usefulness. The consistently high R^2^ values suggest that the model explains most of the observed temporal variability, with remaining residual errors likely attributable to stochastic effects or unmeasured influencing factors (Gandhi et al., 2024).

Despite these improvements, the Vanilla LSTM still exhibits some limitations. For parameters with episodic concentration spikes, such as copper and iron, the uniform temporal weighting applied within the standard LSTM structure may reduce sensitivity to critical historical periods, leading to a slight underestimation of peak concentrations associated with specific operational events.

##### LSTM-attention model performance

The dual-layer LSTM-Attention model achieves the best overall performance across all parameters, showing consistent improvements over the Vanilla LSTM architecture. For arsenic, the MSE decreases from 7.3 × 10^−10^ to 5.6 × 10^−10^, representing a reduction of approximately 23%. Similar performance gains are observed for copper, lead, and zinc.

The improvements are particularly evident for parameters characterised by episodic contamination behaviour. For copper, the MAE is reduced to 0.030 mg/L compared to 0.049 mg/L for the Vanilla LSTM, which is significant given the relatively narrow regulatory threshold of 1.5 mg/L. For suspended solids, the reduction in MSE, although modest in absolute terms, represents an important improvement in prediction accuracy near regulatory limits.

These performance gains are primarily associated with the attention mechanism, which allows the model to dynamically assign greater importance to specific historical time steps. The learned attention weights indicate that recent observations as well as temporally analogous periods play a critical role in shaping predictions. This dynamic weighting improves the model’s ability to capture regime changes and sharp concentration variations, resulting in more reliable forecasts (Wen & Liu, 2023).

The consistently high R^2^ values across all parameters further indicate that the LSTM-Attention model is robust and performs reliably under varying contamination dynamics, in contrast to the ARIMA model, which exhibits parameter-specific failures.

Table 3 demonstrates that the propozed models effectively captured the underlying patterns in the data series. Through rigorous hyperparameter tuning, the LSTM-Attention model achieved superior performance, characterized by significantly lower error metrics (MSE and MAE) and higher coefficients of determination (R^2^ approximatively 0.99) across nearly all parameters compared to the Vanilla-LSTM and the ARIMA baseline (Wang et al., 1993, 2018). Notably, for heavy metals such as As, Ni, and Pb, the LSTM-Attention model maintained high precision where the statistical ARIMA model struggled, as evidenced by the latter’s negative or low R^2^ values. This high predictive accuracy is further validated by the residual analysis presented in Fig. 10. The residuals, which represent the difference between observed and predicted values, confirm the model’s reliability and its ability to minimize systematic bias in effluent concentration forecasting (Da Silva et al., 2025).

#### Residual analysis and model reliability

**Figure. 4:**
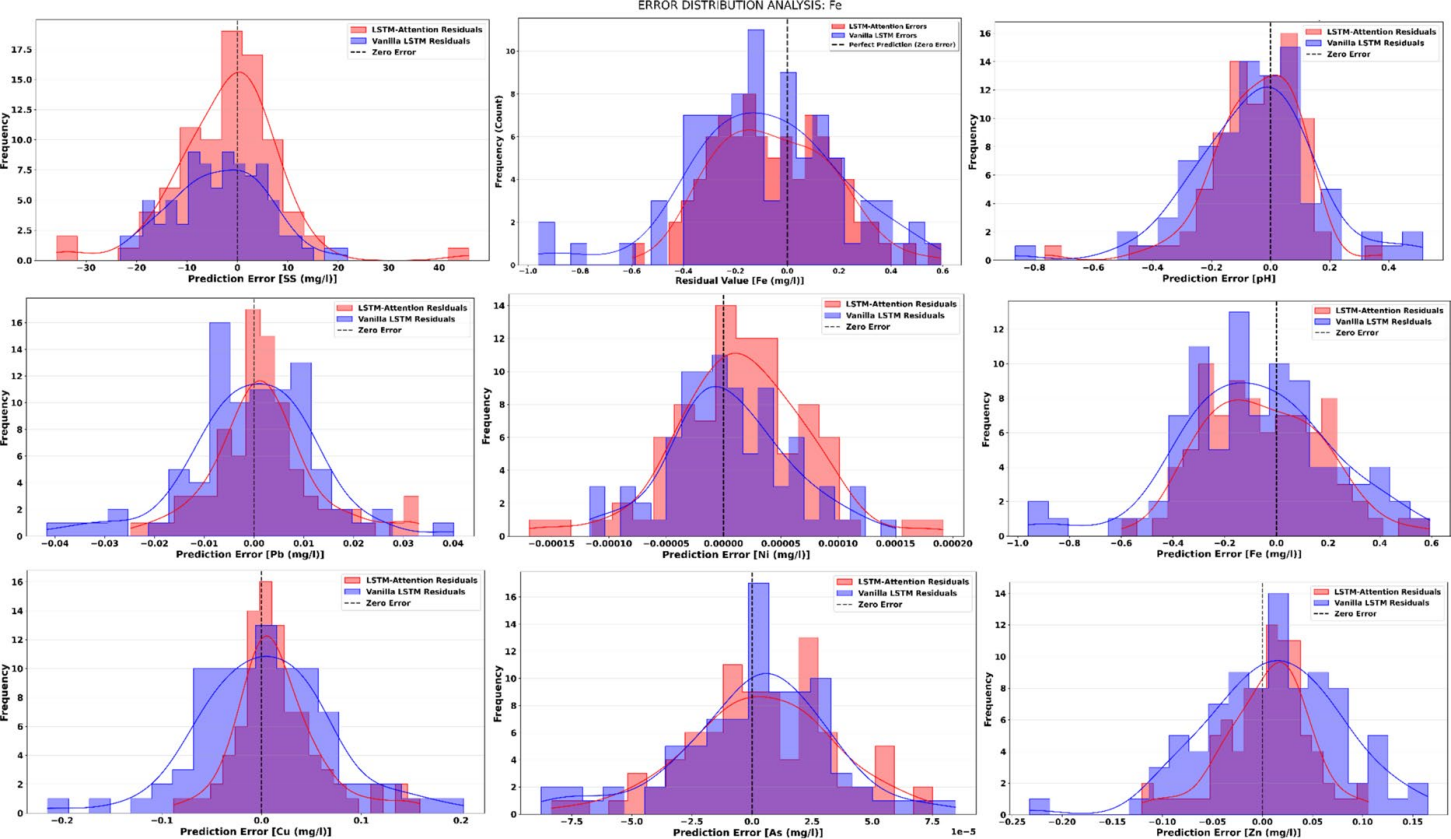
Residual distribution plots for SS, pH, As, Cu, Fe, Zn, Pb, Ni

Residual analysis provides important diagnostic information regarding prediction bias, variance structure, and model reliability. Figure 4 illustrates the residual distributions obtained for all eight parameters using the LSTM-Attention model, with Vanilla LSTM residuals included for comparative purposes. Ideally, residuals should be centered around zero, display an approximately symmetric distribution, and exhibit relatively constant variance across the prediction range (Patthi et al., 2024; Zhang et al., 2021). Arsenic, copper, lead, and suspended solids display well-behaved residual distributions under the LSTM-Attention model. These distributions are approximately normal, centered near zero, and show minimal skewness, indicating the absence of systematic over- or under-prediction. The narrow spread of residuals suggests that prediction errors remain within acceptable limits for operational and regulatory applications. In comparison, Vanilla LSTM residuals exhibit slightly broader tails and minor asymmetry, particularly for copper and iron, indicating that the attention mechanism contributes to reducing occasional large prediction errors.

Iron, zinc, and pH exhibit residual distributions that are generally well centered but show minor deviations from perfect normality. Iron residuals display slight positive skewness, likely reflecting the difficulty in predicting unusually high concentration events. Zinc and pH residuals show marginally heavier tails, indicating a slightly increased occurrence of moderate errors while still maintaining high overall prediction accuracy. Nickel displays the narrowest residual distribution among all parameters, reflecting both the temporal stability of observed nickel concentrations and the strong predictive capability of the LSTM-Attention model. This suggests that nickel forecasts carry relatively low uncertainty compared to other parameters.

ARIMA residuals were excluded from the residual analysis figures, as they do not provide meaningful insights into deep learning model performance. Also, ARIMA residuals exhibit strong deviations from normality and heteroscedasticity for several parameters, which would obscure the comparative analysis between Vanilla LSTM and LSTM-Attention models (Gandhi et al., 2024; Yao et al., 2023).

### Models predictions

**Figure. 5:**
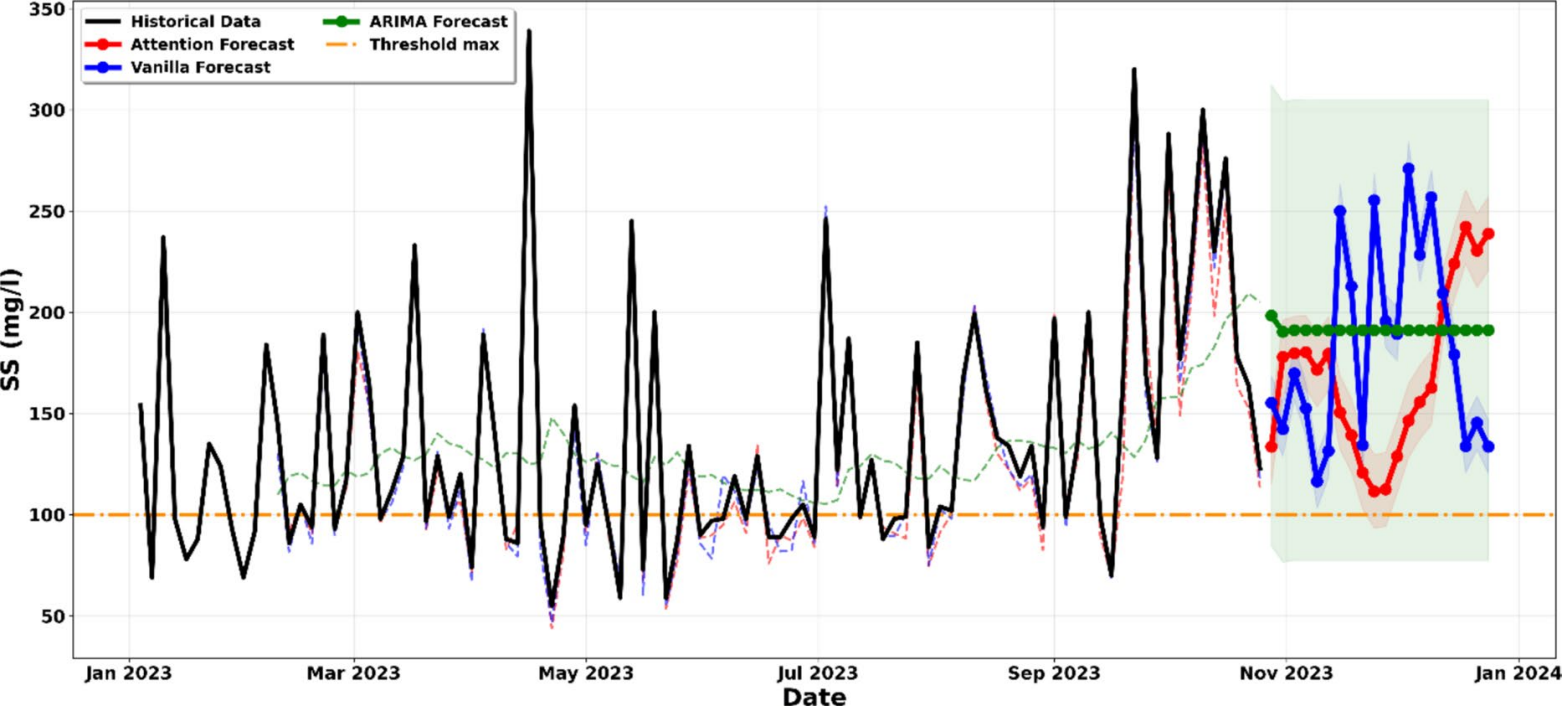
Projection of the evolution of suspended solids until December 2023

**Figure. 6:**
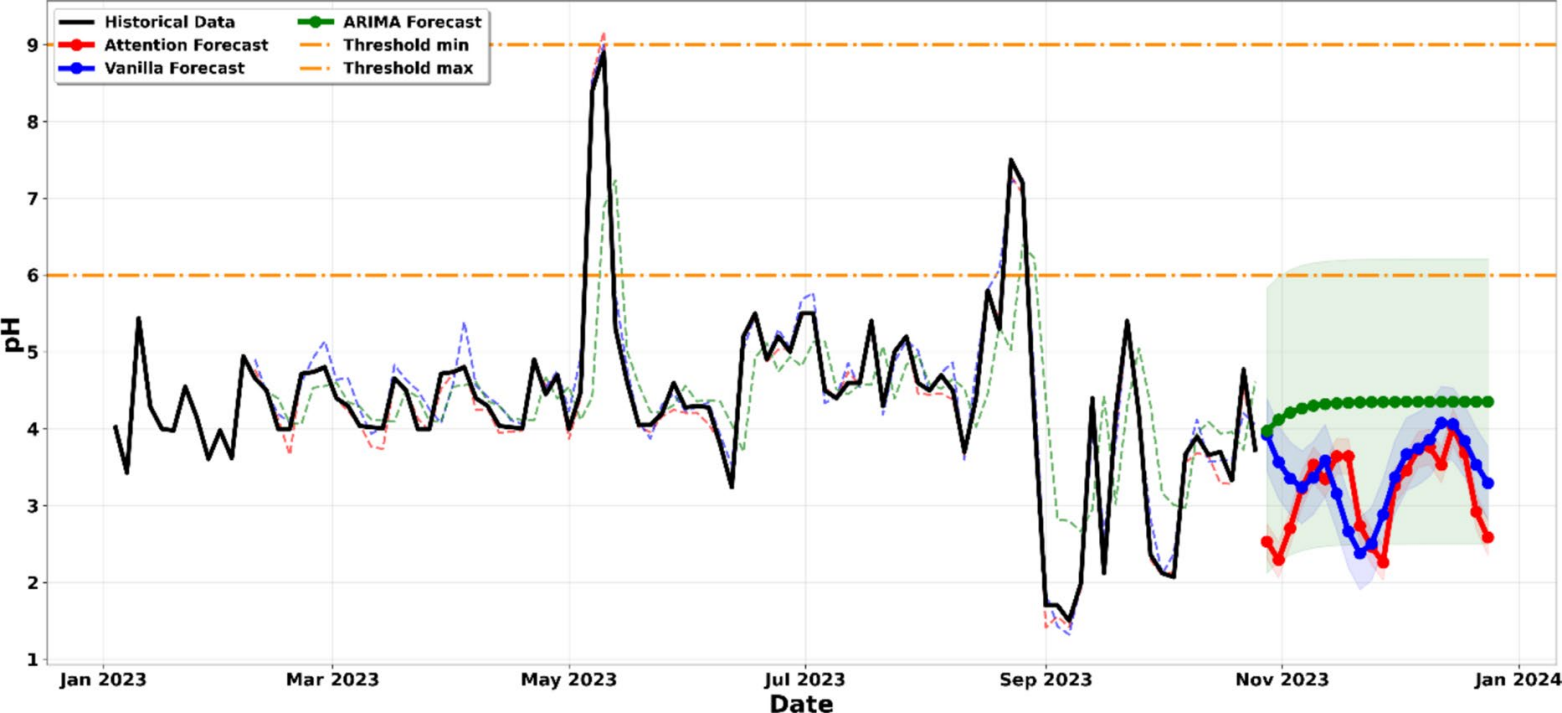
Projection of the evolution of pH until December 2023

**Figure. 7:**
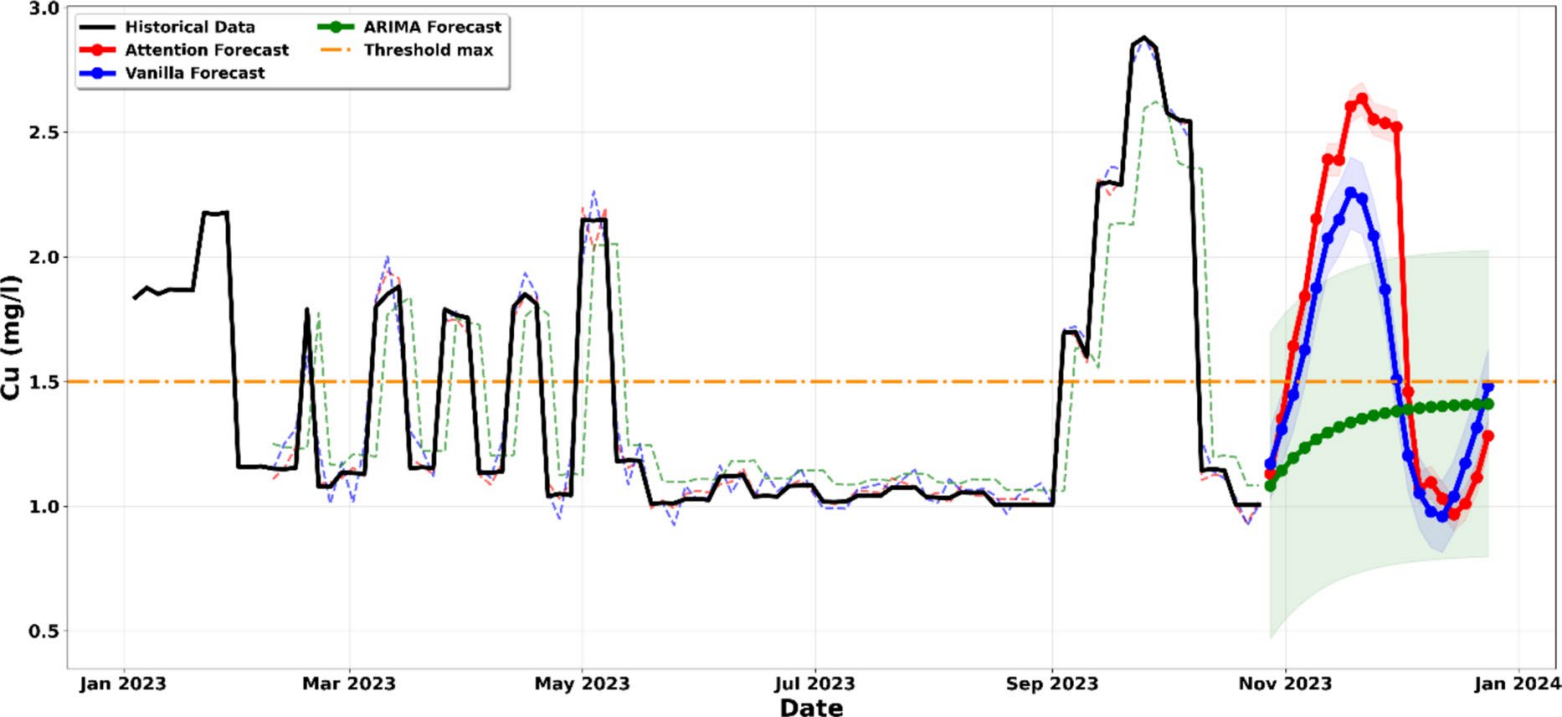
Projection of the evolution of the concentration in Cu until December 2023

**Figure. 8:**
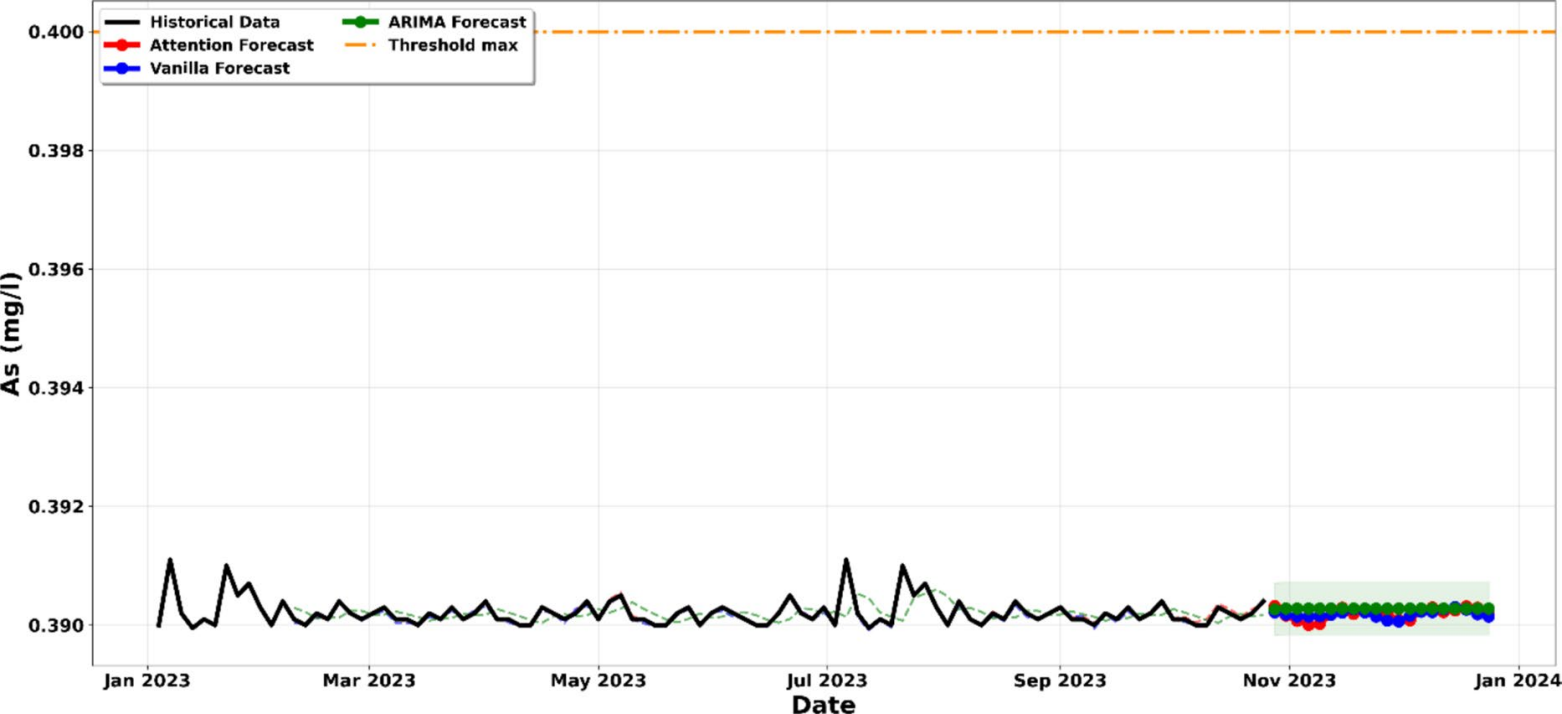
Projection of the concentration of arsenic until December 2023

**Figure. 9:**
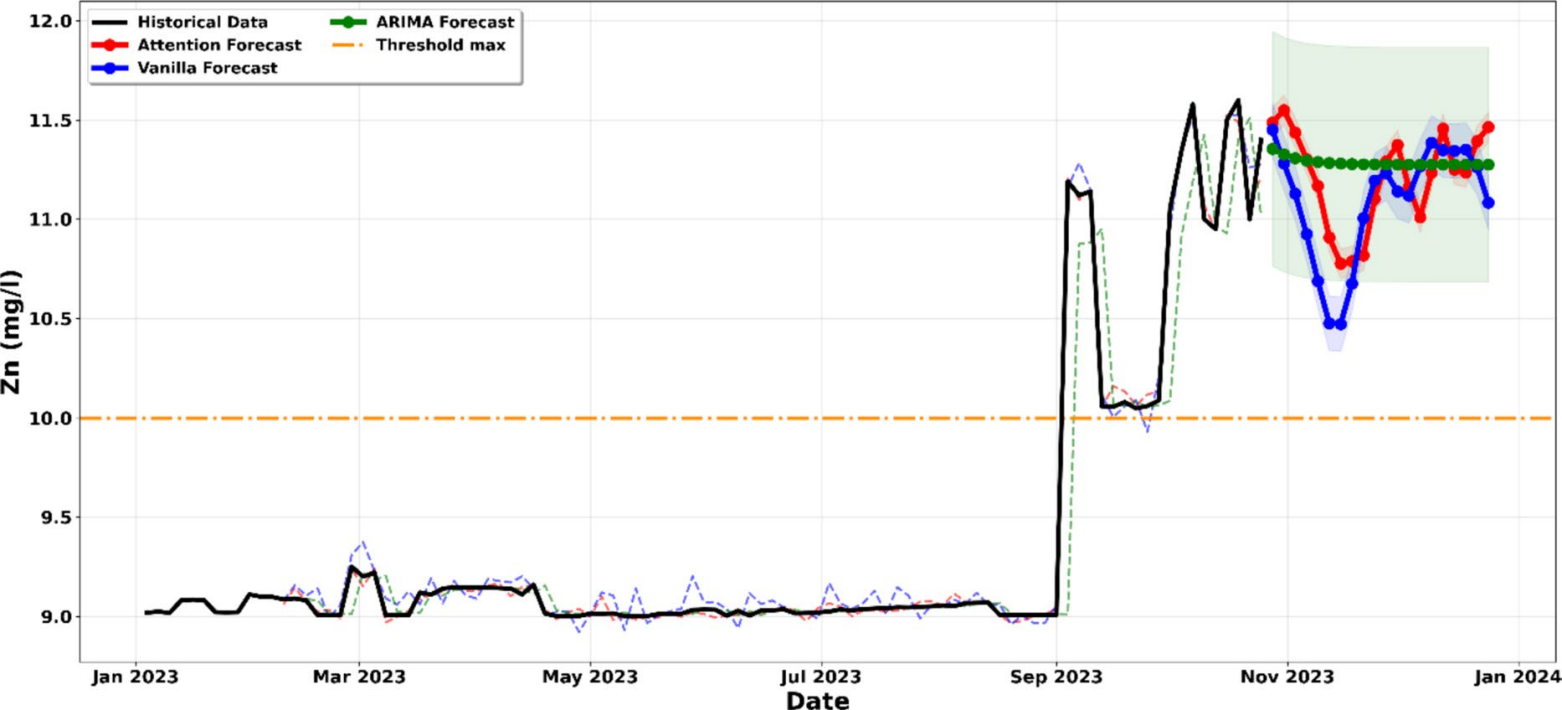
Projection of the evolution of Zinc (Zn) concentration until December 2023

**Figure. 10:**
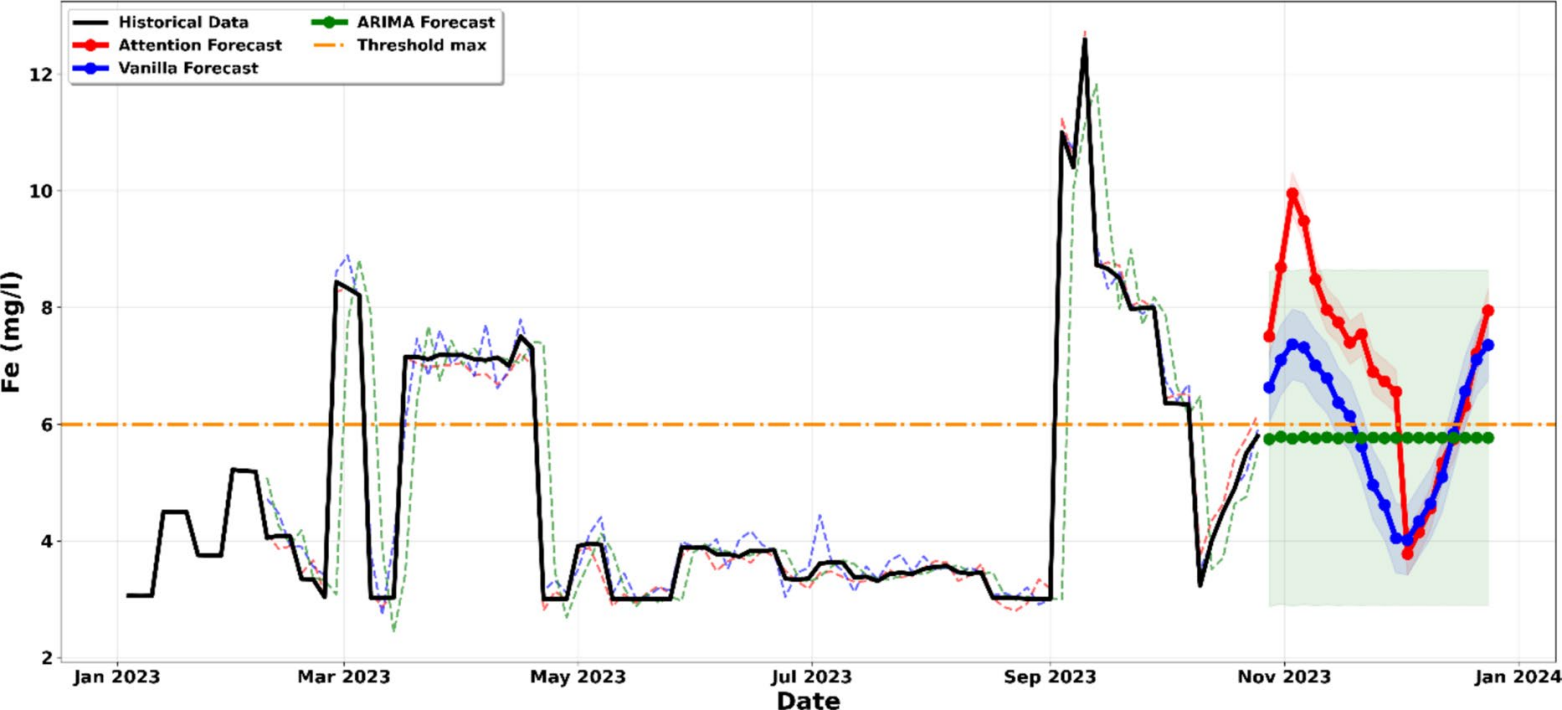
Projection of the evolution of the concentration of iron until December 2023

**Figure. 11:**
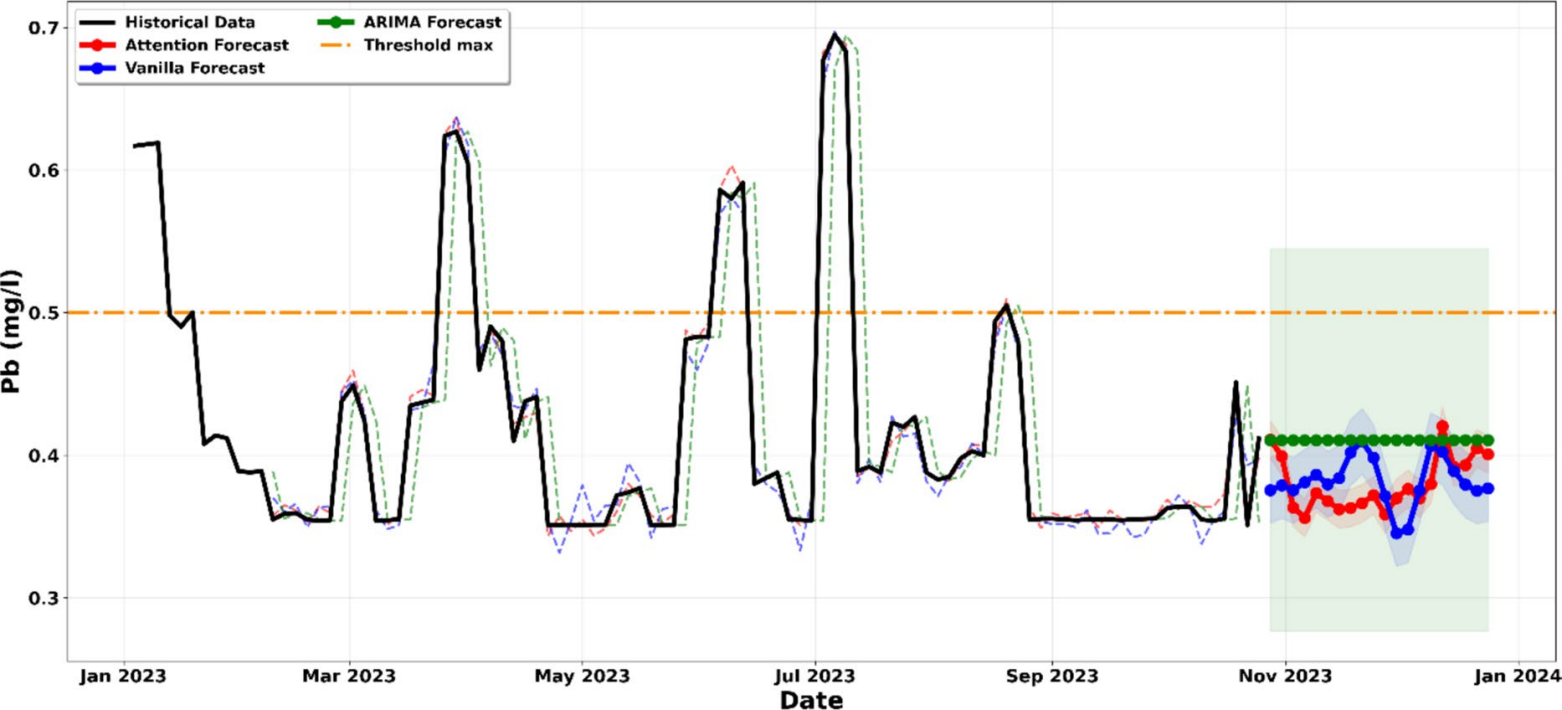
Projection of the evolution of the concentration of lead (Pb) until December 2023

**Figure. 12:**
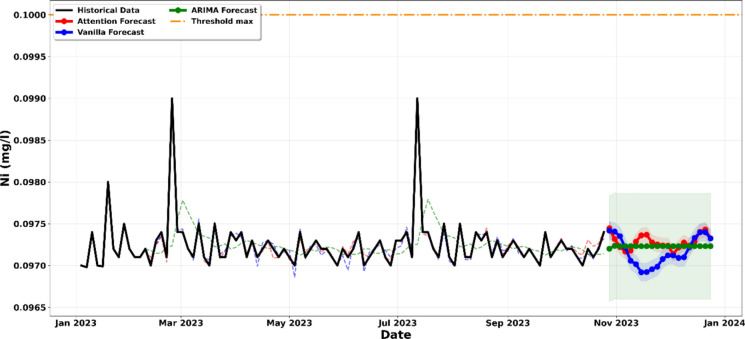
Projection of the evolution of the concentration in Nickel until December 2023

All models generated recursive forecasts of approximately 8 to 10 weeks, starting from the end of the historical dataset in October 2023 and extending through to December 2023. These forecasts incorporate 95% confidence intervals that were calculated as ± 1.96σ, where σ represents the residual standard deviation obtained from the test dataset. The shaded regions illustrated in Figs. 5–12 represent the probabilistic range within which future values are expected to occur with 95% confidence, assuming that operational conditions remain comparable to those observed during the historical monitoring period.

A critical principle for interpretation is applied in this assessment. When the entire 95% confidence envelope, including both the upper and lower bounds, lies either completely above or completely below a regulatory threshold, this is interpreted as a statistically robust indication of noncompliance or compliance, respectively. In cases where the confidence envelope intersects or straddles the regulatory threshold, the forecast is considered uncertain and indicates the need for precautionary management measures rather than definitive conclusions (Kalala et al., 2016; Rahman et al., 2025).

The analysis of Fig. 5 provides a critical warning regarding the concentration of suspended solids in the effluent. An evaluation of the convergence of three predictive architectures, namely ARIMA, Vanilla LSTM, and LSTM Attention, shows a consistent upward trend extending to December 2023. This agreement across models is significant, as the ARIMA model establishes a baseline statistical tendency toward noncompliance, while the LSTM-based models further reinforce this pattern by capturing complex nonlinear dependencies present in the recent data (Salari et al., 2025; Yin et al., 2025). More importantly, the 95% confidence intervals, represented by the shaded regions, indicate that the 100 mg per litre regulatory threshold is not only exceeded by the mean forecasts but also falls well within the statistically probable range of outcomes. Even the best-case scenarios, defined by the lower bounds of these intervals, approach the regulatory limit, indicating that the risk of environmental noncompliance is not simply possible but highly probable and therefore requires immediate operational intervention (Aniyikaiye et al., 2019; Wang et al., 2018).

The longitudinal analysis of Fig. 6 reveals a concerning and persistent acidification trend in the effluent. By leveraging a comparative modeling approach, we observe that the ARIMA, Vanilla LSTM, and LSTM-Attention models all converge on a significant downward trajectory that extends through the end of 2023. While the historical data shows fluctuations, the predictive models indicate that the pH will consistently breach the lower regulatory limit of 6.0 established by the mining code (Koblan et al., 2023; Nwobi et al., 2025). The technical weight of this forecast is reinforced by the 95% confidence intervals; the shaded regions for all three models remain almost entirely below the minimum threshold, suggesting that the probability of the pH returning to a compliant neutral range without intervention is very low. Furthermore, the LSTM-Attention model, which prioritizes recent volatility, suggests an even more aggressive acidification, signaling that the buffering capacity of the receiving environment is at risk of imminent collapse. This cross-model convergence provides a strong indication of sustained non-compliance under current operating conditions (Al-Bahadili & Drewil, 2022; Hu et al., 2025).

The analysis of Fig. 7 reveals a critical and alarming trend toward a severe breach of the 1.5 mg/l regulatory threshold for Copper (Cu) concentrations. The strength of this interpretation is rooted in the convergence of three distinct predictive architectures ARIMA, Vanilla LSTM, and LSTM-Attention all of which confirm an immediate and sharp upward dynamic in November 2023 (Han et al., 2024; Jadhav & Pawar, 2023). The LSTM-Attention model (red line), which excels at capturing complex non-linear dependencies, projects a peak exceeding 2.5 mg/l. This represents a concentration nearly double the authorised limit. The significance of this forecast is further reinforced by the 95% confidence intervals (shaded regions). For the deep learning models, nearly the entire probability envelope sits well above the 1.5 mg/l threshold. This indicates that, statistically, there is a high probability that actual values will exceed the legal limit, even under the most optimistic scenarios. While the ARIMA model (green line) shows a smoother linear progression, the LSTM models capture an aggressive cyclicity, suggesting that these high-pollution peaks are becoming the new operational baseline. This cross-model validation indicates that the current treatment system is no longer capable of stabilizing the metallic load (Liu et al., 2025; Yao et al., 2023). Consequently, the risk of environmental non-compliance is elevated and warrants immediate attention. Immediate corrective intervention in the water treatment process is imperative to prevent a major ecological impact on the receiving environment (Moayedi et al., 2023; Rahman et al., 2025).

The analysis presented in Fig. 8 confirms that Arsenic (As) concentrations remain well within regulatory boundaries, showing no historical or projected trend of non-compliance. A comparative assessment of the ARIMA, Vanilla LSTM, and LSTM-Attention models reveals a stable and consistent trajectory through December 2023, with all three models predicting concentrations significantly below the 0.4 mg/l threshold established by the mining code. The technical reliability of this forecast is further validated by the 95% confidence intervals (Salari et al., 2025). Even the upper boundaries of these shaded probability envelopes remain far below the regulatory limit, indicating a very low statistical probability of an accidental breach under current operating conditions. While the LSTM models capture minor fluctuations in the historical series, the overall stability suggests that the current treatment process is highly effective at managing Arsenic loads. Consequently, the effluent is expected to maintain full environmental compliance for this specific parameter, posing a negligible risk to the receiving aquatic ecosystem (Hu et al., 2025; Kumar & Yadav, 2023).

The data visualised in Fig. 9 confirms a critical and sustained upward trend in Zinc (Zn) concentrations, which are projected to remain consistently above the regulatory threshold of 10 mg/l through December 2025. The high degree of agreement between the ARIMA, Vanilla LSTM, and LSTM-Attention models reinforces the reliability of this forecast, indicating that the observed increase is not a transient fluctuation but a persistent systemic trend (Leščešen et al., 2025; Liu et al., 2025). The strength of this conclusion is validated by the 95% Confidence Intervals (shaded areas); for all three modeling frameworks, the entire probability envelope is positioned significantly above the 10 mg/l limit. This statistical alignment suggests a high likelihood of continued non-compliance, as even the lower bounds of the predicted range fail to descend into the compliant zone. Notably, the LSTM-Attention model suggests that the concentration will not only remain high but may undergo periods of accelerated growth, likely driven by cumulative factors in the effluent discharge. This cross-model convergence signals an urgent need for technological adjustments in the zinc recovery or precipitation stages, as the current treatment infrastructure is statistically indicated to be insufficient for meeting mining code standards (Chen et al., 2018; Olascoaga & Haller, 2012).

The data presented in Fig. 10 indicate that iron concentrations in the laboratory liquid effluents remain a persistent compliance concern, as values consistently exceed the regulatory threshold of 6 mg per litre. The convergence of the ARIMA, Vanilla LSTM, and LSTM Attention models reveals a highly volatile pattern, which is typical of laboratory-generated waste where effluent quality varies according to the type of mineral samples processed and the reagents applied during analysis. This interpretation is further strengthened by the LSTM Attention model, shown by the red curve, which captures a projected peak approaching 10 mg per litre in November. This behavior suggests that specific laboratory cycles, likely associated with intensive mineral digestion or high-iron sample batches, periodically exceed the capacity of the existing effluent management system (Al-Janabi et al., 2020; Korunoski, 2019).

The 95% confidence intervals, represented by the shaded regions, provide strong statistical evidence of this risk, as even the lower bounds remain largely above the 6 mg per litre regulatory limit, indicating that the likelihood of compliance under current laboratory practices is statistically minimal. These results imply that the downward trends observed at certain periods reflect temporary reductions in laboratory activity rather than sustained improvements in effluent quality. The continued positioning of the probability envelope above the threshold confirms a high probability of discharging non-compliant effluent, which necessitates targeted laboratory level interventions, such as enhanced neutralisation processes or selective precipitation of iron from the analytical waste stream, to ensure compliance with the mining code before discharge into the receiving environment (Chen et al., 2018; Runge & Zmeureanu, 2021).

The analysis of Fig. 11 indicates that Lead (Pb) concentrations currently maintain a fragile compliance with the 0.5 mg/l regulatory threshold. While the historical data and the primary forecasts from the ARIMA, Vanilla LSTM, and LSTM-Attention models suggest that the mean concentration will remain below the limit through late 2023, the margin of safety is remarkably narrow. The strength of this interpretation lies in the evaluation of the 95% Confidence Intervals (shaded regions). Although the solid trend lines remain below the threshold, the upper boundaries of the probability envelopes for both the LSTM-Attention and Vanilla LSTM models frequently touch or cross the 0.5 mg/l line. Statistically, this implies that even a minor variation in laboratory operating conditions such as the analysis of lead-rich galena samples or a slight change in the acid digestion protocols, could increase the risk of threshold exceedance (Hu et al., 2025; Moayedi et al., 2024).

Unlike other parameters where the breach is certain, Lead represents a ‘high-sensitivity’ variable. The convergence of the models suggests that while the laboratory is not currently in a state of chronic failure for this metal, it lacks the industrial buffer to absorb any increase in analytical workload or reagent intensity. Consequently, Fig. 11 serves as a proactive warning: without strict waste-stream segregation or pre-treatment of high-lead batches, the laboratory is one operational fluctuation away from a mining code violation.

The data presented in Fig. 12 confirms that Nickel (Ni) concentrations in the laboratory effluents are characterised by high stability and consistent compliance with the 1 mg/l regulatory threshold. The comparative analysis using ARIMA, Vanilla LSTM, and LSTM-Attention models demonstrates a sustained horizontal trajectory, indicating that Nickel is not currently a high-risk pollutant. The technical strength of this conclusion is anchored in the 95% confidence intervals (shaded areas). Across all three forecasting frameworks, the entire probability envelope, including the most pessimistic upper bounds, remains significantly below the 1 mg/l limit. This provides strong statistical evidence that even with the inherent variability of mineral analysis cycles, the risk of a Nickel breach before the end of 2023 is very low (Jadhav & Pawar, 2023; Li et al., 2025). Unlike the volatile trends observed in Iron or Copper, the Nickel series suggests that either the mineral samples processed have low Nickel content or the laboratory’s precipitation protocols are exceptionally efficient for this specific metal. Consequently, Fig. 12 illustrates a state of robust compliance, where the current waste management system provides an ample safety buffer. This allows the laboratory to focus its immediate remedial efforts on more critical parameters like SS or pH, while maintaining a simple monitoring protocol for Nickel to ensure this stable trend continues (Omisore, 2018; Wen & Liu, 2023).

## Discussion

### Environmental contamination risks

The predictive assessment indicates the presence of a broader environmental compliance failure that extends beyond isolated exceedances of individual parameters. Before discussing these findings in detail, it is important to acknowledge dataset constraints: the monitoring period covers n = 43 weekly observations from a single laboratory site. The high R^2^ values (≈ 0.99) must be interpreted in light of this limited temporal scope; weekly aggregation smooths short-term fluctuations, which may reduce apparent model complexity and inflate goodness-of-fit metrics. All findings should therefore be treated as site-specific and exploratory, requiring multi-site replication before broader conclusions are drawn. The combined occurrence of persistent acidification conditions (pH 2.8–3.5), elevated suspended solids concentrations (150–220 mg/L), and multiple heavy metal exceedances (Cu, Zn, Fe) results in a complex contamination setting in which synergistic toxicity effects are likely to occur, exceeding the impacts expected from individual pollutants acting independently. Low pH conditions enhance the mobilisation of heavy metals from sediment matrices, thereby increasing their bioavailability and associated toxicity. Previous studies have shown that copper toxicity to fish can increase by up to tenfold when pH decreases from neutral conditions (pH 7) to acidic environments (pH 4), largely due to the increased activity of free Cu^2+^ ions, which represent the most toxic copper species. In a similar manner, iron under pH values lower than 4 promotes the mobilisation of aluminium from clay minerals, leading to aluminium toxicity that damages fish gill tissues and interferes with osmoregulatory processes (Hu et al., 2025; Kumar & Yadav, 2023).

The forecasted high particulate concentrations (136–220 mg/L) also act as transport media for adsorbed heavy metals, organic substances, and other associated contaminants. These particles tend to accumulate in depositional environments such as slow-flowing river sections and floodplain areas, where they form long-term contamination sinks. Benthic organisms ingest these contaminated particles, resulting in metal bioaccumulation and subsequent transfer through the aquatic food web to fish and, ultimately, to human populations relying on fish consumption from the impacted watershed.

Considering an estimated effluent discharge rate of approximately 15–20 m^3^/day, derived from flow measurements, and assuming discharge into a small receiving stream with a base flow of 50–100 L/s, the initial dilution factor is expected to be limited to approximately 3–5 times. This restricted dilution capacity implies that pollutant concentrations at distances of 100–500 m downstream would remain well above regulatory thresholds for pH (pH 4.5–5.0), copper (0.5–1.0 mg/L), and suspended solids (40–70 mg/L). As a result, the zone of significant environmental impact is estimated to extend several kilometres downstream, affecting riverside communities that depend on the water for domestic use, agricultural activities, and fishing practices. These estimates are indicative only, derived from simplified dilution modelling, and require validation through formal hydrodynamic studies (e.g., tracer experiments, river flow gauging, numerical hydrodynamic modelling) before being used for regulatory planning (Moayedi et al., 2024).

### Comparison with previous research

The findings obtained are consistent with, and also extend, earlier investigations of mining-related contamination within Central Africa. Kalala et al. (2016), for example, reported comparable pH exceedances (pH 3.2–4.8) and elevated copper concentrations (1.1–2.3 mg/L) associated with effluent discharges from the STL smelter into the Lubumbashi River. These similarities indicate that chronic acidification represents a widespread and systemic issue across multiple mining and metallurgical operations in Haut-Katanga Province, rather than an isolated or site-specific occurrence.

However, the present study advances previous research in several important aspects. It provides a time-resolved predictive evaluation of laboratory-scale effluents, demonstrating that even relatively small discharge volumes can contribute substantially to cumulative contamination at the watershed scale. In addition, the integration of deep learning-based forecasting with regulatory compliance analysis enables the generation of quantitative probability estimates, expressed through 95% confidence intervals, rather than relying solely on qualitative risk descriptions. Furthermore, the development of parameter-specific risk stratification (Table 4) allows for prioritisation of targeted mitigation measures instead of broadly defined recommendations to improve treatment performance (Moayedi et al., 2023; Rahman et al., 2025).

**Table 4:** Risk Prioritisation Matrix for Monitored Parameters

Parameter	Exceedance Probability (%)	Environmental Toxicity	Priority Tier
pH	> 90% (below 6.0)	High (acidification, metal mobilisation)	CRITICAL
SS	~ 85%	Moderate (carrier for metals)	CRITICAL
Cu	~ 82%	High (aquatic toxicity at low concentrations)	CRITICAL
Zn	~ 91%	Moderate–High (chronic aquatic toxicity)	CRITICAL
Fe	~ 75%	Moderate (aesthetic, indirect effects)	HIGH
Pb	~ 74%	High (cumulative, no safe threshold)	HIGH
As	< 5%	High (carcinogenic)	MONITORING

### Model performance

The comparative modelling framework applied in this study yielded several methodological insights that are relevant for environmental forecasting applications beyond the specific case considered. The consistent superior performance of LSTM-based models compared to ARIMA across all analysed parameters, with R^2^ improvements ranging from 0.20 to 0.60, indicates that laboratory effluent contamination processes exhibit inherently nonlinear temporal behaviours that conventional statistical approaches are not able to adequately represent. This observation aligns with recent findings reported in the environmental forecasting literature (Gandhi et al., 2024; Leščešen et al., 2025) and suggests that LSTM-based approaches are more appropriate for pollution prediction in complex industrial and laboratory environments.

Additional performance gains observed for the LSTM-Attention model relative to the Vanilla LSTM, reflected by MSE reductions between 23 and 71%, were particularly evident for parameters characterised by episodic contamination events, such as copper, iron, and lead. This pattern provides empirical support for the value of attention mechanisms in situations where pollution signals are temporally irregular, which is commonly observed in laboratory-based and batch-process effluents. In contrast, for parameters displaying smoother temporal trends or relatively stable behaviour, the additional benefit of attention mechanisms appears limited, suggesting that model complexity should be aligned with data characteristics (Wen & Liu, 2023).

The residual distributions associated with the LSTM-Attention predictions were close to ideal. However, the risk of overfitting cannot be dismissed given the small dataset (n = 43). To address this, two validation strategies were applied. First, training and validation loss curves were examined and showed convergence without significant divergence across epochs, indicating the absence of classical overfitting during training. Second, rolling forecasting origin cross-validation (RFOCV) was implemented as a time-series-appropriate evaluation method. In this forward-chaining scheme, the model was trained on an expanding window beginning with a minimum training set of 22 observations (approximately 50% of the dataset), with the origin advanced one step at a time; at each origin, predictions were generated for a one-step-ahead horizon and evaluated against the held-out observation. This procedure preserves the chronological ordering of the data, eliminates data leakage, and prevents future observations from informing predictions of earlier time points. Across the 21 rolling evaluation windows, mean R^2^ values remained consistent with those of the dedicated hold-out test set (within ± 0.03 across all parameters), and mean absolute errors were comparable to those reported in Table 3. These results provide additional confidence that the reported performance metrics reflect genuine generalisation rather than artefacts of validation design, though the inherently limited sample size means that caution remains warranted in interpreting all findings as site-specific and exploratory. The absence of systematic bias, heteroscedastic patterns, or strong deviations from normality supports the application of confidence intervals for probabilistic forecasting, which is an essential requirement for risk-informed environmental management and regulatory decision-making (Patthi et al., 2024; Zheng et al., 2025). Although the 20-week forecasting horizon demonstrated acceptable predictive accuracy, preliminary extensions to longer horizons of 6–12 months resulted in rapidly increasing uncertainty, limiting their practical usefulness for decision-making. This suggests that recursive forecasting approaches are appropriate for short- to medium-term operational planning horizons, but should not be applied to longer-term strategic planning without incorporating additional contextual information.

### Implications for sustainable development goals

The outcomes of this research can be directly linked to specific United Nations SDG sub-indicators. The modelled probabilities of Cu exceedance (~ 82% of forecast weeks above 1.5 mg/L), Zn exceedance (~ 91% of weeks above 10 mg/L), and Pb exceedance (~ 74% of weeks above 0.5 mg/L) constitute direct empirical evidence of chronic pollution discharge. These findings connect to SDG sub-indicator 6.3.2 (proportion of water bodies with good ambient water quality) and to the SDG 6.3 target of ‘reducing pollution, eliminating dumping and minimising release of hazardous chemicals and materials’. The predictive early-warning framework operationalises this target by enabling proactive intervention before exceedances occur, rather than relying on retrospective compliance checks. For SDG 3.9 (substantially reducing mortality and illness from hazardous chemicals and air, water, and soil pollution by 2030), the cadmium and lead exceedance findings are particularly relevant: both metals bioaccumulate in the food chain, and predicted receiving-stream concentrations after conservative dilution remain above WHO drinking-water guideline values, posing direct risks to downstream communities. The parameter-specific risk-prioritisation matrix (Table 4) provides a concrete pathway toward more responsible management of analytical reagents and process chemicals in line with SDG 12 (Target 12.4: responsible management of chemicals throughout their life cycle). Downstream transport through the Congo River network also implies potential long-range implications for SDG 14 (Life Below Water), although formal hydrological modelling is needed to quantify this pathway.

## Limitations and future perspective

This study acknowledges several important limitations related to data availability, model structure, and operational variability. Dependence on existing datasets restricts the detection of sudden pollution events, while the temporal resolution of sampling may overlook short lived contamination episodes, which can reduce prediction accuracy and lower confidence in short term model forecasts (Moayedi et al., 2024). In addition, focusing exclusively on Haut Katanga in the Democratic Republic of Congo limits the generalisability of the findings, as effluent characteristics may differ under different mining practices, laboratory protocols, seasonal production cycles, and regulatory frameworks (Muniz et al., 2025; Patthi et al., 2024).

Although these modelling methods are advanced, they also have limitations. The LSTM Attention model, while capable of producing accurate predictions, is computationally complex and prone to overfitting, particularly when applied to highly nonlinear environmental data (Shakhsher et al., 2014; Yin et al., 2025). Model performance is further influenced by unmodelled factors, including seasonality of production, variations in laboratory work intensity, and differences in analytical procedures, which introduce measurement uncertainty. As a result, the forecasts largely represent extrapolation of historical behaviour rather than definitive prediction of future pollution threats. In addition, common statistical metrics such as R^2^ and MSE, although useful, do not fully capture the multi-dimensional nature of environmental compliance and pollution risk (Aniyikaiye et al., 2019).

Future research should address these limitations through continuous monitoring systems with higher temporal resolution, enabling real time detection of sudden contamination events (Leščešen et al., 2025; Rouessac et al., 2004). Incorporating additional explanatory variables, such as seasonal production data, meteorological conditions, upstream environmental factors, and laboratory operational parameters, has strong potential to improve model robustness and predictive reliability. Exploring ensemble and hybrid modelling approaches may further enhance accuracy. Regular evaluation of treatment technologies remains essential for improving laboratory operations, ensuring regulatory compliance, and supporting the United Nations sustainability goals through proactive protection of aquatic ecosystems and effective resource management (Hu et al., 2025; Kumar & Yadav, 2023).

## Conclusion

The predictive analysis of the liquid effluents targeted in this study reveals environmental realities demanding immediate attention amid current ecological and climatic challenges. Implementing a high-precision LSTM-Attention modeling approach, this research advances beyond simple observation to deliver a quantitative assessment of future contamination risks. The model’s reliability was confirmed by a very high coefficient of determination (R^2^ > 0.99) and minimal prediction errors (MSE), establishing a solid foundation for long-term forecasting through the end of 2023.

The integration of 95% confidence intervals into the forecasts provided strong empirical evidence of probable non-compliance for several critical parameters. Given the limited dataset (n = 43 weekly observations, single site), these findings represent high-likelihood risk indications rather than deterministic predictions. While the laboratory effluents currently show stable and compliant levels for Arsenic and Nickel, an elevated and persistent risk of threshold exceedance was identified for Suspended Solids (SS), Copper, Zinc, and Iron. For these contaminants, the full probability envelope remains above the thresholds established by the DRC Mining Regulations, demonstrating that current management practices are inadequate to prevent environmental degradation. Also, the predicted persistent acidification of effluent pH, combined with the narrow safety margin for Lead, indicates that even minor operational variations in laboratory procedures, such as the digestion of metal-rich ore samples, could increase the risk of regulatory threshold exceedance.

To achieve effective environmental protection, a specialised treatment hierarchy for laboratory waste streams is essential. This should include alkaline neutralisation to address chronic acidity (average pH 4.35), advanced filtration to substantially reduce suspended solids concentrations, and targeted demineralisation or chemical precipitation to remove metallic elements prior to discharge. Given the high statistical probability of threshold exceedance forecasted by the models, these interventions are no longer optional but essential for the laboratory to maintain its social and legal license to operate within the Haut-Katanga Province.

The findings confirm the effectiveness of the proposed approach for the specific mineral analysis laboratory studied in the Haut-Katanga Copperbelt. All conclusions are site-specific and exploratory; multi-site replication across laboratories with different analytical profiles, ore types, and operational contexts is required before this framework can be considered broadly applicable. The results offer practical guidance for regulators and laboratory managers at the study site, directly supporting SDG 6.3 (reducing water pollution and eliminating dumping), SDG 3.9 (reducing illness from pollution), and SDG 12.4 (responsible chemical lifecycle management), while identifying priorities for future multi-site research and real-time monitoring.

## Supplementary Information

Below is the link to the electronic supplementary material.

Supplementary file1 (DOCX 20 kb)

## Data Availability

Data will be made available on request.
