# Supplementary material for: Predictive Modeling of Mining Laboratory Effluent Contamination Using LSTM-Attention Networks: A Case Study from the Haut-Katanga Copperbelt
Source: Environ Geochem Health. 2026 Jun 3;48(9):386. doi: 10.1007/s10653-026-03262-0 (PMC13233990; doi:10.1007/s10653-026-03262-0)
Supplement: Supplementary file 1 — Supplementary file1 (DOCX 20 kb) [file 10653_2026_3262_MOESM1_ESM.docx]

# **SUPPLEMENTARY MATERIAL**

### **ARIMA Model Order**

Table S1 presents the selected ARIMA order for each parameter, the corresponding AIC value, the Ljung-Box test result at lag 10, and the interpretation of residual adequacy. Note that while ARIMA (1,1,1) was optimal for the majority of parameters, five parameters (Fe, Zn, Pb, As, Ni) required different specifications reflecting their distinct temporal structures.

| **Parameter** | **Selected Order (p, d, q)** | **p** | **d** | **q** | **AIC** | **Ljung-Box p-value (lag 10)** | **Residual Adequacy** |
| --- | --- | --- | --- | --- | --- | --- | --- |
| **pH** | **ARIMA (1,1,1)** | 1 | 1 | 1 | −28.4 | 0.312 | Residual diagnostics indicate no significant autocorrelation at any tested lag, suggesting an adequate model fit. |
| **SS** | **ARIMA (1,1,1)** | 1 | 1 | 1 | 312.7 | 0.284 | Residual analysis shows minor positive skewness, attributable to the October peak; this deviation is considered acceptable for the baseline model. |
| **Cu** | **ARIMA (1,1,2)** | 1 | 1 | 2 | −62.1 | 0.198 | Model adequacy is confirmed, with the additional moving average (MA) term capturing episodic copper spikes that were not resolved by the MA (1) specification. |
| **Fe** | **ARIMA (2,1,1)** | 2 | 1 | 1 | 118.3 | 0.241 | Model adequacy is confirmed for copper, with AR (2) required to capture two-week lagged autocorrelation arising from batch digestion cycles. |
| **Zn** | **ARIMA (1,1,0)** | 1 | 1 | 0 | −44.8 | 0.403 | Model adequacy is confirmed for zinc, which exhibits a near-monotonic trend; inclusion of the MA term provides no significant improvement. |
| **Pb** | **ARIMA (0,1,1)** | 0 | 1 | 1 | −98.6 | 0.267 | Model adequacy is confirmed for lead, which exhibits near-random walk behavior; the autoregressive component is not significant. |
| **As** | **ARIMA (0,0,0)** | 0 | 0 | 0 | −312.4 | 0.891 | Model adequacy is confirmed for arsenic, which remains effectively constant (0.390 mg/L throughout); a white noise model is therefore considered optimal. |
| **Ni** | **ARIMA (0,1,1)** | 0 | 1 | 1 | −287.3 | 0.512 | Model adequacy is confirmed for nickel, which exhibits very low variability; MA (1) after differencing adequately captures the residual structure. |

Table S1. Per-parameter ARIMA order selection results. AIC = Akaike Information Criterion (lower = better fit); Ljung-Box p-value > 0.05 indicates no significant residual autocorrelation at lag 10; SS = Suspended Solids.

Table (S1) reports the results of the ARIMA (Autoregressive Integrated Moving Average) model order selection procedure applied independently to each of the eight monitored effluent quality parameters. As explained in Section 2.7.1 of the main manuscript, ARIMA models were employed as a classical statistical baseline rather than as direct competitors to the deep learning architectures (Vanilla LSTM and LSTM-Attention). Their purpose is to provide an interpretable reference point against which the incremental predictive gain of neural network-based approaches can be quantified.

For each parameter independently, the optimal ARIMA order (p, d, q) was determined through an exhaustive grid search over the following parameter space:

- Autoregressive order: p ∈ {0, 1, 2, 3}
- Differencing order: d ∈ {0, 1, 2}
- Moving-average order: q ∈ {0, 1, 2, 3}

The selection criterion was the Akaike Information Criterion (AIC), which penalises model complexity relative to goodness of fit and is widely used for ARIMA order identification in environmental time-series analysis. The AIC is defined as:

*AIC = 2k − 2ln(L̂)*

where k is the number of estimated parameters and L̂ is the maximised likelihood of the model. Lower AIC values indicate a better balance between model fit and parsimony.

Stationarity was assessed prior to fitting using the Augmented Dickey-Fuller (ADF) test. Parameters requiring differencing to achieve stationarity (ADF p-value > 0.05) were assigned d ≥ 1. Residual model adequacy was verified post-fitting using the Ljung-Box portmanteau test (tested at lags h = 10 and h = 20), which evaluates the null hypothesis that residuals are independently distributed (i.e., no remaining autocorrelation structure). A Ljung-Box p-value > 0.05 confirms adequate model fit.

### **Per-Parameter Data** **Summary**

Table S2 provides a comprehensive summary of the raw data characteristics for each of the eight monitored effluent quality parameters

| **Parameter** | **Measurement Frequency** | **Total Raw Observations** | **Aggregation Method** | **Weekly Observations (n)** | **Missing Values (%)** | **Outliers Removed (n)** | **Notes on Data Quality** |
| --- | --- | --- | --- | --- | --- | --- | --- |
| **pH** | 3×/week (Mon, Wed, Fri) | 130 | Weekly mean of 3 measurements | **43** | 1.5% (2 values) | 3 | 2 missing values due to pH electrode failure in September. 3 outliers removed: single anomalous readings inconsistent with adjacent measurements, traced to calibration drift. |
| **SS** | 3×/week (Mon, Wed, Fri) | 130 | Weekly mean of 3 measurements | **43** | 0.8% (1 value) | 2 | 1 missing value (February equipment maintenance). 2 outliers: one traced to formazin standard contamination; one to sample handling error. |
| **Cu** | 1×/week (composite) | 43 | Direct use of composite | **43** | 0.0% (0 values) | 1 | No missing values. 1 outlier (September, 3.87 mg/L) reviewed against the laboratory logbook and retained as genuine, representing a peak during the analytical campaign |
| **Fe** | 1×/week (composite) | 43 | Direct use of composite | **43** | 2.3% (1 value) | 0 | 1 missing value: composite sample lost during transport (broken bottle). No statistical outliers detected; September peak (8.69 mg/L) confirmed as real event. |
| **Zn** | 1×/week (composite) | 43 | Direct use of composite | **43** | 0.0% (0 values) | 2 | No missing values. 2 outliers removed: both traced to Zn contamination of blank solutions during AAS calibration, confirmed by blank re-analysis. |
| **Pb** | 1×/week (composite) | 43 | Direct use of composite | **43** | 0.0% (0 values) | 1 | No missing values. 1 outlier removed: single value 10× above adjacent observations; traced to sample mislabelling (high-Pb galena digest). |
| **As** | 1×/week (composite) | 43 | Direct use of composite | **43** | 4.7% (2 values) | 0 | 2 missing values: both due to graphite furnace AAS maintenance requiring furnace replacement. No outliers were identified for arsenic, which exhibited near-zero variability throughout the monitoring period. |
| **Ni** | 1×/week (composite) | 43 | Direct use of composite | **43** | 2.3% (1 value) | 0 | 1 missing value due to multi-element standard depletion. No outliers detected; Ni concentrations stable throughout monitoring period. |

Table S2. Per-parameter data summary for all eight monitored effluent quality parameters (January–October 2023). SS = Suspended Solids; AAS = Atomic Absorption Spectrometry; IQR = Interquartile Range. Missing value percentages are calculated relative to the expected total of 43 weekly observations. Yellow highlighting indicates the parameter with the highest missing data rate (As: 4.7%). All missing values were treated by linear interpolation before modelling.

Missing values in the dataset arose from two sources: (i) occasional equipment failures or maintenance requiring postponement of sampling sessions; and (ii) analytical failures, including spectrophotometer calibration drift or sample contamination requiring rejection of results. Missing values were identified by cross-referencing measurement records with laboratory logbooks.

Gap lengths of one week or less were treated by linear interpolation between the preceding and following valid observations, which is appropriate when the gap is sufficiently short that the interpolated value lies within the expected range of natural weekly variability. For gaps extending beyond one week (not observed in this dataset), forward-filling from the last valid observation would have been applied. All missing value treatments were documented in the laboratory logbook and are reflected in the percentage reported in Table S2.

Outlier detection was performed using the Interquartile Range (IQR) method, a non-parametric approach that does not assume a specific distributional form and is robust to the heavy-tailed distributions commonly observed in environmental monitoring data. An observation x was flagged as an outlier if it fell outside the interval [Q1 − 1.5 × IQR, Q3 + 1.5 × IQR], where Q1 and Q3 are the first and third quartiles and IQR = Q3 − Q1.

All flagged observations were individually reviewed against the original laboratory records to distinguish true measurement errors (equipment malfunction, transcription errors, calibration failures) from genuine extreme events. Only observations confirmed as measurement errors were removed; genuine extreme values associated with documented intensive digestion campaigns or equipment anomalies were retained as valid data. Removed values were replaced by linear interpolation, documented in the missing value count, and are included in the missing value percentage in Table S2.
